# Flexible Multi‐Mode Electrochromic Displays for Human Motion Monitoring and Pattern Display in Dark Environments

**DOI:** 10.1002/EXP.20240444

**Published:** 2026-03-16

**Authors:** Xue Chen, Qi Zhao, Li Wang, Xin Yu, Xu Liang, Zhang Chen, John Wang, Yanfeng Gao

**Affiliations:** ^1^ State Key Laboratory of Advanced Refractories Shanghai University Shanghai China; ^2^ Department of Materials Science and Engineering National University of Singapore Singapore Singapore

**Keywords:** flexible multi‐mode electrochromic display, monitor human motion, optical adaptive display

## Abstract

Flexible electrochromic displays (ECDs) demonstrate low energy consumption, good readability, and high color contrast. However, their performance deteriorates in dark environments due to the reliance on environment light. Herein, we propose a flexible multi‐mode electrochromic display (FMECD), which is designed to effectively integrate electrochromism‐photoluminescence‐mechanoluminescence (EC‐PL‐ML) functionalities. The PL and ML serve as backlight to simulate a natural light condition for display in dark environment. The function is realized by incorporating tannic‐acid‐modified polydimethylsiloxane (PDMS), incorporated with ZnS: Cu phosphor as a backlight, onto which patterned tungsten trioxide (WO_3_)‐EC devices are applied. The FMECD shows a large optical modulation of 75.8% and maintains robust EC performance with a reflection retention of 86% after 200 stretching cycles, and 91.5% after 500 bending cycles. Notably, compared to commercial organic light‐emitting diode (OLED) displays, the FMECD significantly reduces energy consumption from 172.2 Wh m^−2^ to 0.0075 Wh m^−2^. As a proof of concept, we demonstrate the functionality of the FMECD as a patterned display that visualizes various motion states of different body parts in multiple scenarios of dark environments, advancing the development of wearable intelligent displays for human motion monitoring and signal patterning.

## Graphical abstract

The reflective nature of electrochromic displays limits their display functionality and human motion monitoring in dark environments. This work presents a flexible multi‐mode electrochromic display (FMECD) with high optical modulation (75.8%) and low energy consumption (0.004% of OLEDs). Integrating EC, PL, and ML functions, it enables continuous, real‐time motion display in complex environments, offering a promising solution for wearable intelligent displays.

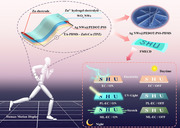

## 1 Introduction

Wearable displays, known for their lightweight and adaptive properties, have received considerable attention in the field of human‐machine interfaces [1, 2, 3, 4]. Flexible liquid crystal display (LCD), organic light‐emitting diode (OLED) and electrochromic displays (ECDs) can effectively convert various physical, chemical, and biological signals [5, 6, 7, 8], into visual patterns for monitoring human motion [9, 10, 11, 12]. Among them, LCD and OLED displays currently dominate the market [13, 14, 15]. However, the continuous static display requires a constant power supply to operate OLED, leading to significantly high power consumption and accelerated device aging [16, 17]. ECDs offer the unique advantage of low energy consumptions [18, 19, 20, 21, 22]. They use an electric field to switch the color of material, requiring minimal power for static display. Particularly, they can maintain high visibility for more than 2 h without external power [23]. Therefore, the selection of electrochromic materials as fundamental structural units is advantageous for the fabrication of displays with low energy consumption and high color fidelity.

To date, in dark environments and under flexible operating conditions, ECDs struggle with patterned display due to their reflective nature [24, 25, 26, 27, 28]. Specifically, ECDs rely on environment light that reflects off their surfaces to induce visible color changes. In low‐light or dark environments, the absence of sufficient external light severely diminishes the visibility of electrochromic patterns, thereby limiting their display capability [29]. Furthermore, when operating in flexible configurations, the inherent changes in surface geometry and light reflection characteristics can further exacerbate the problem, leading to a diminished display quality. In such case, integrating with a luminescent functionality, such as electrochemical luminescence (ECL), electrofluorescence discoloration (EFC), and photoluminescence (PL), represents an effective strategy. Recent studies have demonstrated that devices combining electrochromic (EC) with PL or EFC materials can achieve a reflection/emission dual mode for pattern display in dark environments [25, 30, 31, 32, 33]. However, these devices require additional energy input from ultraviolet light‐emitting diode (UV LED) backlights or other power sources, limiting their large scale applications [34, 35, 36]. Differentiated from these strategies, mechanoluminescence (ML) converts mechanical stimuli, such as bumping, bending, squeezing, impinging, scanning, stretching, wind, and raindrops, into light, effectively reducing the reliance on external energy sources [37, 38, 39]. ML emission triggered by tensile deformation is particularly useful in dark environments.

Despite these advances, current research on multimodal display systems remains in its infancy. Most efforts focus on dual‐mode integrations, such as EC–PL or EC–EFC, aiming to enhance visibility under variable lighting [8, 30, 35]. The simultaneous integration of EC, PL, and ML functionalities into a unified platform has rarely been explored. Furthermore, most existing studies evaluate device performance under static or idealized laboratory conditions [4, 34, 40, 41]. However it is necessary to investigate in real‐world environments involving mechanical stress, temperature fluctuations, or dynamic motion (Table S1). These gaps hinder the practical deployment of flexible displays in wearable electronics, where multimodal responsiveness and environmental robustness are critical.

**FIGURE 1:**
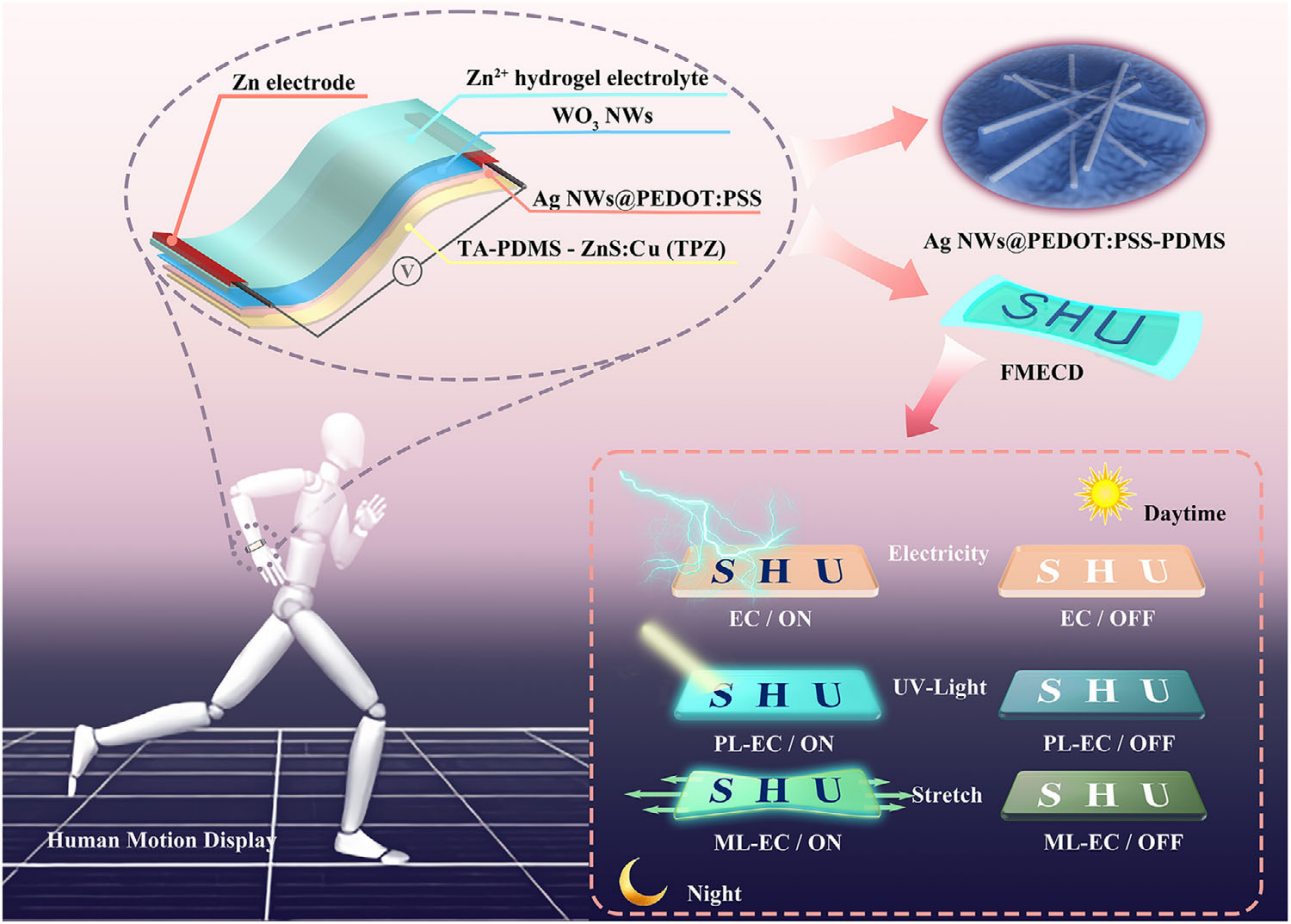
Structure and multi‐mode functional display application of FMECDs, which consist of multiple layers: Ag NWs are sprayed onto TA‐modified PDMS (TA‐PDMS) substrate as the conductive electrode. A polymer poly(3,4‐ethylenedioxythiophene) polystyrene sulfonate (PEDOT: PSS) is coated on it to form Ag NWs@PEDOT: PSS flexible conductive layer. Patterned WO_3_ is then sprayed on Ag NWs@PEDOT: PSS as an electrochromic active layer. TA‐PDMS with mixed ZnS: Cu phosphor (TPZ) as backlight, Ag NWs@PEDOT: PSS as the flexible conductive layer, WO_3_ as the electrochromic layer, Zn^2+^ hydrogel as the electrolyte, and zinc electrode as the counter electrode are integrated in FMECDs. FMECDs can thus realize the display function in EC, EC‐PL and EC‐ML modes in light/dark environments.

We have designed a flexible multi‐mode electrochromic display (FMECD) that integrates the EC‐PL‐ML technologies together, for applications in complete darkness environments. The new design emphasizes substrate compatibility, multimodal display functionality, stability in complex environments, and is targeted wearable applications. The configuration and operational features of FMECDs are shown in Figure 1. Tannic acid (TA) modification is employed to reduce the hydrophobic mismatch between polydimethylsiloxane (PDMS) and silver nanowires (Ag NWs). Simultaneously, tungsten trioxide (WO_3_) is purposely sprayed on the PDMS for pattern design, and combined with PL‐ML backlight substrate incorporating ZnS: Cu phosphors to achieve multimodal patterned displays. Compared to commercial OLED, this new integration allows for low‐energy consumption FMECDs, effectively reducing energy use from 172.2 Wh m^−2^ to 0.0075 Wh m^−2^ (0.004%). Consequently, the FMECD demonstrates excellent stretchability and EC performance, retaining 86% and 91.5% of the EC efficiency after 200 times of stretching and 500 times back and forth 180 degrees of bending cycles, respectively. For multidisciplinary applications, the FMECD integrates with sensors to monitor and display human motion states by detecting and analyzing the intensity and frequency of luminescence signals.

## 2 Results and Discussion

### 2.1 Structural Optimization and EC Performance for FMECDs

**FIGURE 2:**
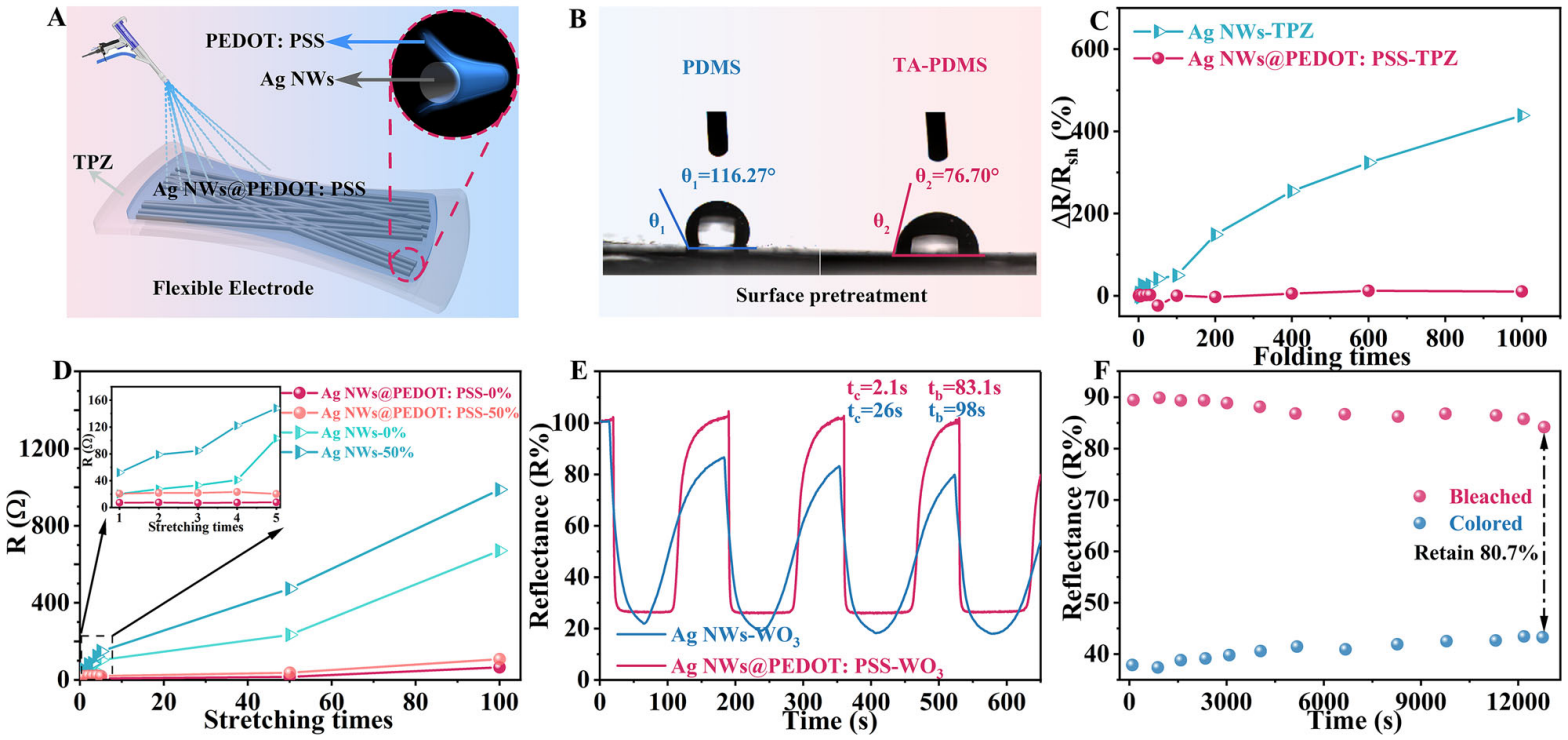
Electrical conductivity and EC properties of FMECDs. (A) Schematic diagram of flexible electrode structure in FMECDs, TPZ is a flexible substrate for TA modified PDMS mixed ZnS: Cu phosphors. (B) Water infiltration Angle of untreated PDMS and TA‐PDMS substrates. Resistance changes of Ag NWs‐TPZ and Ag NWs@PEDOT: PSS‐TPZ conductive substrate during (C) bending and (D) stretching tests. (E) In‐situ reflection spectra of FMECDs at 633 nm. (F) Cyclic stability of FMECDs.

FMECDs are fabricated using WO_3_ film as the EC display layer, Zn^2+^ hydrogel as the electrolyte, Ag NWs@PEDOT: PSS as the flexible conductive layer, and ZnS: Cu phosphor‐mixed PDMS (TPZ) as the flexible substrate (Figure 1 and Figure 2A). Polydimethylsiloxane (PDMS) is super‐hydrophobic, and TA modification will enhance the attachment of Ag NWs on PDMS (Figure S1, Supporting Information). The FTIR results of TA‐PDMS and Ag NWs indicate that TA has been coated on the surface of PDMS. Hydrogen bonds can be formed between the hydroxyl group in TA‐PDMS and the amide group in Ag NWs (Figure S2a and S2b, Supporting Information). The scanning electron microscope (SEM) images and EDS mappings results further verified that the presence of TA significantly enhanced the adhesion of Ag NWs to PDMS. The adsorption capacity of Ag NWs to PDMS increases from 0.12% to 28.78% (Figure S2c and S2d, Supporting Information). In addition, the surface modification of PDMS by TA effectively transforms the PDMS surface from hydrophobic to hydrophilic (Figure 2B, contact angle reduced from 116.27° to 76.70°).

PEDOT: PSS has excellent electrical conductivity, flexibility, and chemical stability [21, 42, 43]. It is selected as the protective coating layer, encapsulating the entire Ag NWs network (Figure S3, Supporting Information), which can effectively improve the mechanical properties and environmental tolerance of Ag NWs. Based on the micromorphology studies, PEDOT: PSS is known to evenly distribute on the PDMS substrate and forms a core‐shell structure with Ag NWs (Figure S4, Supporting Information). Ag NWs‐TPZ and Ag NWs@PEDOT: PSS‐TPZ demonstrate repeatable performance under repeated stretching and bending cycles, with the comparative results presented in Figure 2C,D. The resistance of the Ag NWs‐TPZ conductive substrate increases approximately three times after 1,000 times of 180° folding. Upon 100 cycles of repeated 50% stretching, the resistance increases to 100 times of its initial value. In contrast, the Ag NWs@PEDOT: PSS‐TPZ conductive substrate exhibits only a 10% increase in resistance after 1000 folding cycles. Similarly, after 100 cycles of repeated stretching, the resistance only increases from the initial 10 Ω to 100 Ω. In addition, compared to other commonly reported flexible transparent electrodes (Table S3), the Ag NWs@PEDOT: PSS electrode developed in this work exhibits clear advantages.

The observed results can be attributed to the enhanced stability provided by the network structure of Ag NWs. Additionally, the incorporation of PEDOT: PSS serves to protect the force‐bearing junctions of the Ag NWs, preventing the fracture of the nanowires. The supplementary PEDOT: PSS polymer forms a stable and efficient conductive pathway between the silver nanowires in localized nanoscale regions during the stretching process [43]. Consequently, the resistance change of the Ag NWs@PEDOT: PSS‐TPZ film remains minimal under external mechanical stress. Comparably, the resistance of commercial ITO/PET increases rapidly after bendings (Figure S6, Supporting Information) of 187 times (1.51 kΩ). In addition, the resistance of the Ag NWs@PEDOT: PSS‐TPZ conductive substrate is rather stable for more than 8 days, increasing by only 4%, while the Ag NWs‐TPZ conductive substrate increases by up to 60.3% (Figure S7, Supporting Information). This core‐shell structure effectively mitigates the oxidation of the Ag NWs electrode during the electrochromic process of the FMECD, ensuring a sustainable EC process (Figure S5, Supporting Information). Therefore, the Ag NWs@PEDOT: PSS electrode achieves an optimal balance among conductivity, flexibility, and mechanical robustness, making it highly suitable for application in flexible electrochromic devices (Table S2, Supporting Information).

The FMECD with PEDOT: PSS turns dark blue at ‐1.0 V, and transits to a transparent state at +1.0 V, indicating a charging and discharge process, respectively (Figure S8, Supporting Information). The switching speeds, defined by the time to achieve 90% of an entire optical change, are 2.1 s for coloring and 83.1 s for bleaching, which are notably faster than those of the FMECD without PEDOT: PSS (Figure 2E, 26 s for coloring and 98 s for bleaching). Similarly, the coloring efficiency (CE value, refer to Equation S1, Supporting Information) of 132.2 cm^2^ C^−1^ for the FMECD significantly exceeds that of the device without PEDOT: PSS (43.9 cm^2^ C^−1^, Figure S9, Supporting Information). This much enhanced performance indicates that PEDOT: PSS improves the conductive sites of Ag NWs, compensating for the low conductivity of WO_3_ and thus facilitating electron transfer. In addition, Figure 2F shows the In‐situ reflection spectrum, obtained when the cycle stability of FMECDs is investigated. Upon 200 cycles, the modulation amplitude is 80% of the initial, benefiting from the effective protection of the Ag NWs electrode by PEDOT: PSS. The EC stability and electrochemical performance of FMECDs without PEDOT: PSS protection are suboptimal. As depicted in Figure S10 and S11 (Supporting Information), without the PEDOT: PSS protection, FMECDs completely failed upon 45 cycles of coloring/bleaching [44]. As illustrated in Figure S12 (Supporting Information), In‐situ reflection spectra and cyclic voltammetry curves demonstrate that the presence of PEDOT: PSS makes WO_3_ with more active sites, reflecting a wider light modulation amplitude in optical performance (ΔR_Ag NWs_≈ 52.5%, ΔR_Ag NWs@PEDOT: PSS_≈ 65.2%). The non‐linear shape of the galvanostatic charge‐discharge curves show that Ag NWs@PEDOT: PSS‐WO_3_ electrode has typical pseudocapacitive behavior. When the current density is set at 0.02 mA cm^−2^, the area capacitance of Ag NWs@PEDOT: PSS‐WO_3_ electrode (621 mF cm^−2^) is greater than that of Ag NWs‐WO_3_ electrode (410 mF cm^−2^).

### 2.2 Optical Adaptive FMECDs

**FIGURE 3:**
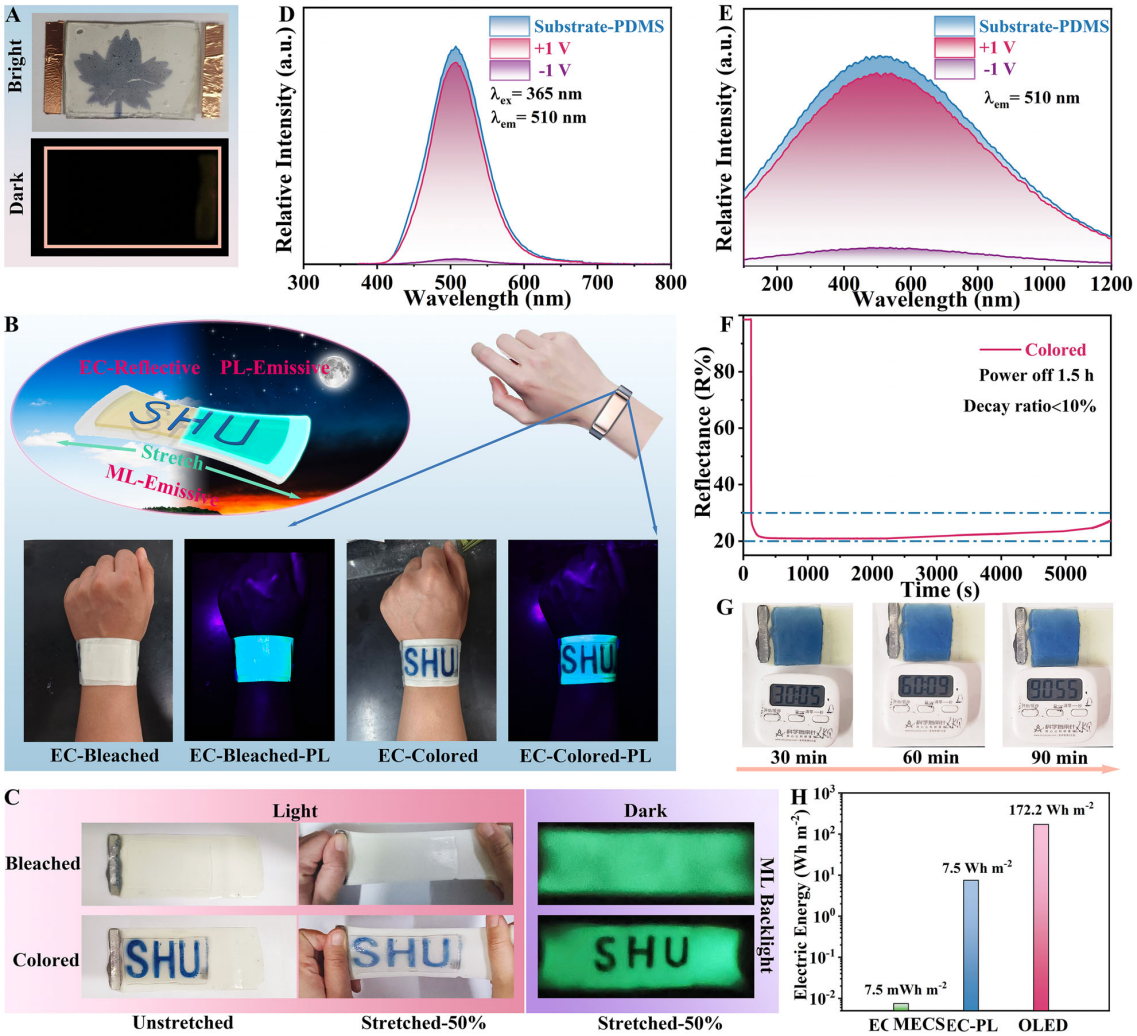
Display function and energy‐saving performance of optical adaptive FMECDs. (A) Display picture of traditional flexible EC devices in bright/dark environment, and the flexible conductive substrate is PET/ITO. (B) Concept demonstration of patterned display of FMECDs. A physical demonstration of the bracelet in EC and EC‐PL modes. (C) Photos for FMECDs EC‐ML mode display. The modulation amplitude of the luminescent intensity of (D) PL‐emission spectrum and (E) ML‐emission spectrum in the “on/off” state. At −1 V stimulation for 70 s, power off for 1.5 h, (F) reflectance and (G) photos of FMECDs. (H) Energy saving calculation results of FMECDs and OLED devices.

ECDs using PET as the flexible substrate are ineffective in the completely dark environment (Figure 3A). Through the pattern design of FMECDs, the display function of EC‐PL‐ML can be made to possess three modes under bright/dark environments. As a conceptual demonstration, we assemble FMECDs with the pattern of “SHU” (5 cm×10 cm and 2 cm×6 cm) as illustrated in Figure 3B,C. In the bright environment, the reflection mode dominated by EC shows that the “on” and “off” states through applied bias. In the dark environment, the PDMS substrate combined with ZnS: Cu phosphor powder shows high PL and ML under UV LED and tensile stress‐strain excitation, serving as an effective backlight for the emission mode, while EC dominated the control of pattern display. In the “on” colored state, the low transmittance of EC film can block the light transmittance of the substrate in the visible spectrum, facilitating a complementary display in the PL and ML emission modes. In the “off” bleached state, the pattern display becomes invisible. Figure 3D,E illustrate that both the PL‐emission and ML‐emission modes exhibit significant changes in the modulation of luminous intensity. In the bleached state, the luminescent intensity of the substrate remains relatively unchanged, indicating that the prepared EC electrode film has no great influence on the luminescent intensity of the substrate backlight. After coloring, the light signal becomes nearly undetectable, showing that most of the light is blocked by EC electrode absorption, indicating an optimal display state. It is worth noting that the reflectivity of FMECDs after coloring can maintain color retention for 1.5 h without the application of any voltage (Figure 3F,G). In addition, the excitation of ML mode requires only mechanical stresses such as compression and stretching. Quantitatively, the energy consumption of FMECDs (0.0075 Wh m^−2^) can be reduced to 0.004%, compared to OLED displays (172.2 Wh m^−2^) over a duration of 1.5 h in dark (Equation S3, Figures S13 and S14, Supporting Information), as shown in Figure 3H. In contrast, the PL mode relies on ultraviolet excitation, resulting in an energy‐saving efficiency that is 4.4% lower than that of OLED displays.

### 2.3 EC Performance of FMECDs in Deformation and Cold Conditions

**FIGURE 4:**
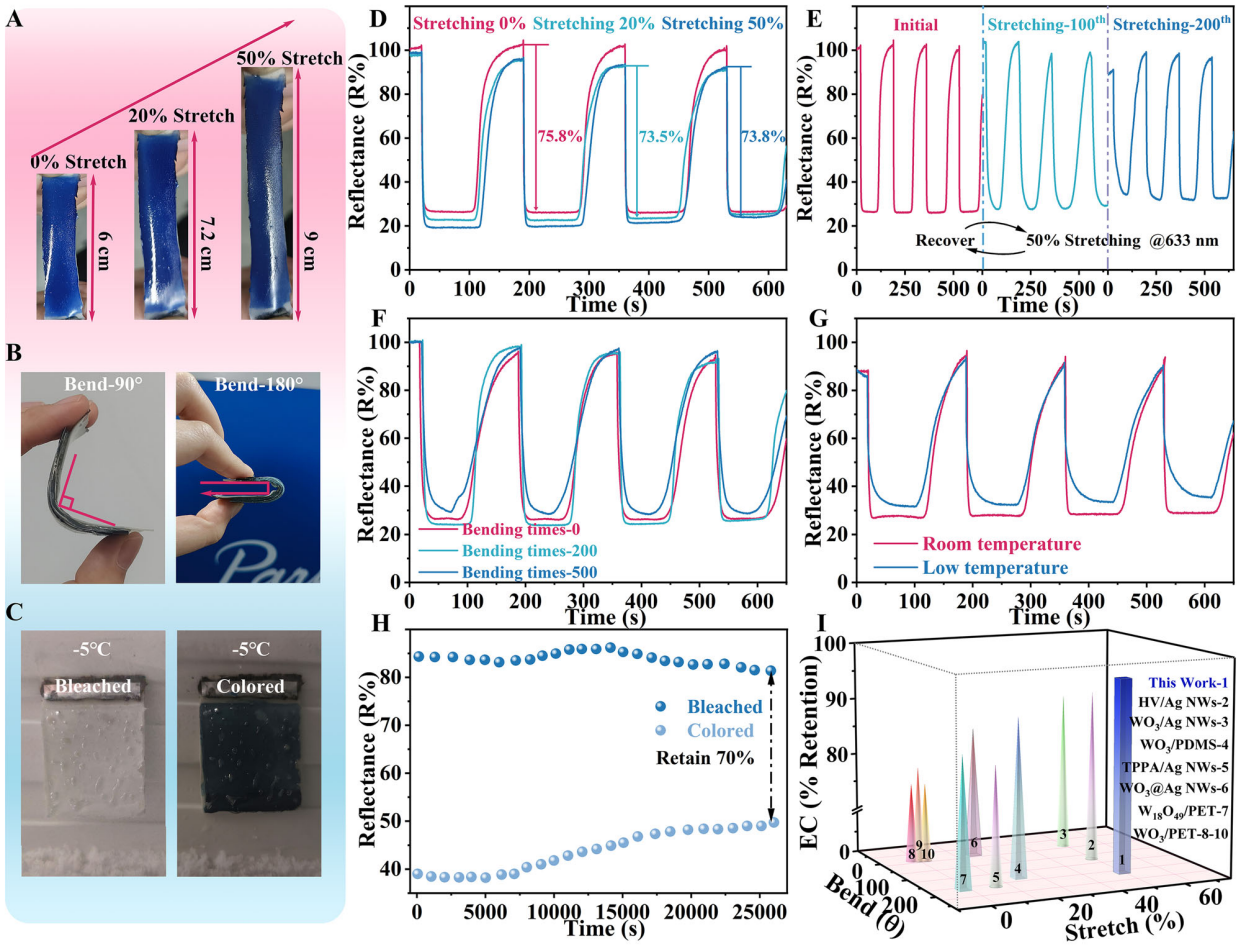
Characterization of EC properties of FMECDs under deformation and at low temperature. (A–C) Test images of the device undergoing 20% and 50% stretching deformation, 180° bending, at −5°C). (D) In‐situ reflectance spectra of FMECDs under 20% and 50% stretching deformation. In‐situ reflectance spectra of FMECDs after (E) repeated stretching (50% strain) for 200 cycles, and (F) bending (180°) for 500 cycles. (G) Low temperature (−5°C) reflection spectrum, and (H) the cycle stability of FMECDs. (I) The results of this work are compared with other reported work on EC retention (The X‐axis denotes the tensile strain (%), the Y‐axis corresponds to the bending angle, and the Z‐axis indicates the retained electrochromic reflectance under mechanical deformations).

To further validate the feasibility of FMECDs in potential application environments, we have evaluated FMECDs in deformation and cold conditions. The initial state of the FMECDs is normalized to 100% as the baseline (Figure S15, Supporting Information), and a wavelength of 633 nm is chosen to examine the switching behavior. Figure 4A–C show the flexibility and EC ability of FMECDs under different bending, stretching, and low temperature states. FMECDs exhibit excellent electrochemical stability at deformation, room temperature, and low temperature, as shown in Figure S16. The corresponding reflectivity modulation amplitude of FMECDs under 20% and 50% tensile deformation shows the variation of less than 5% (Figure 4D). This minor change confirmed the role of a robust flexible structure. This design effectively mitigates the issue of the WO_3_ dark blue coloration fading under straining, thereby preserving display contrast during stretching [45, 46]. The reflectivity of FMECDs was tested for 200 cycles of repeated stretching (50% strain) and 500 cycles of repeated bending (180°) to verify the stability of their electrochromic (EC) performance (Figure 4EF). Upon repeated stretching, the reflection retention rate is approximately 86% of the initial value, with the coloring time increasing to 5.6 s from 2.1 s for the unstretched device. Similarly, upon repeated bendings, the reflection retention rate is about 91.5% of the initial value, with the coloring time extending to 13 s. Although the increase in electrode resistance slows the EC switching speed, FMECDs still maintain the normal display functionality during both repeated stretching and bending.

For practical applications, low‐temperature operation is essential for electrochromic displays, as it enables reliable performance under cold conditions, thereby broadening their potential in outdoor and wearable electronics [34, 47]. To further evaluate the anti‐freezing properties of FMECDs in potential practical applications, the feasibility of FMECDs at low temperatures is verified by low temperature reflection spectrum and cycle stability (Figure 4G,H). At the low temperature of ‐5°C, the modulation amplitude still remains at 70% of the initial value, and the coloring time only increases to 8 s. In addition, Supplementary movie 1 shows the electrochromic effect of FMECD in 95% humidity conditions. Supplementary movie 2 shows the electrochromic effect of FMECD at 80°C. It is important to note that the FMECD works well even in high humidity and temperature. These results show the excellent stability and durability of each FMECD (Figure S17).

Moreover, for comparison purposes, we also conducted In‐situ reflection spectrum tests on the devices without PEDOT: PSS protective layer, as shown in Figures S18–S21 (Supporting Information). The device fails after 200 cycles of repeated stretching, the reflection retention drops to approximately 53% after 500 cycles of bending repeats, and the coloring time is 11.9 s at ‐5°C. A summary of the state‐of‐the‐art flexible electrochromic devices is presented in Figure 4I, which includes WO_3_/PDMS (ΔR retention rate after stretching ≈ 81%), W_18_O_49_/PET (ΔR retention rate after bending 75%), WO_3_/PET (low temperature coloring time 11.4 s), etc [21, 34, 40, 41, 42], under similar experimental conditions (refer to Tables S3–S6, Supporting Information). By comparison, our optimized FMECDs exhibit superior color retention after repeated deformation and enhanced stability even at low temperature.

### 2.4 Multifunctional Display of FMECDs

**FIGURE 5:**
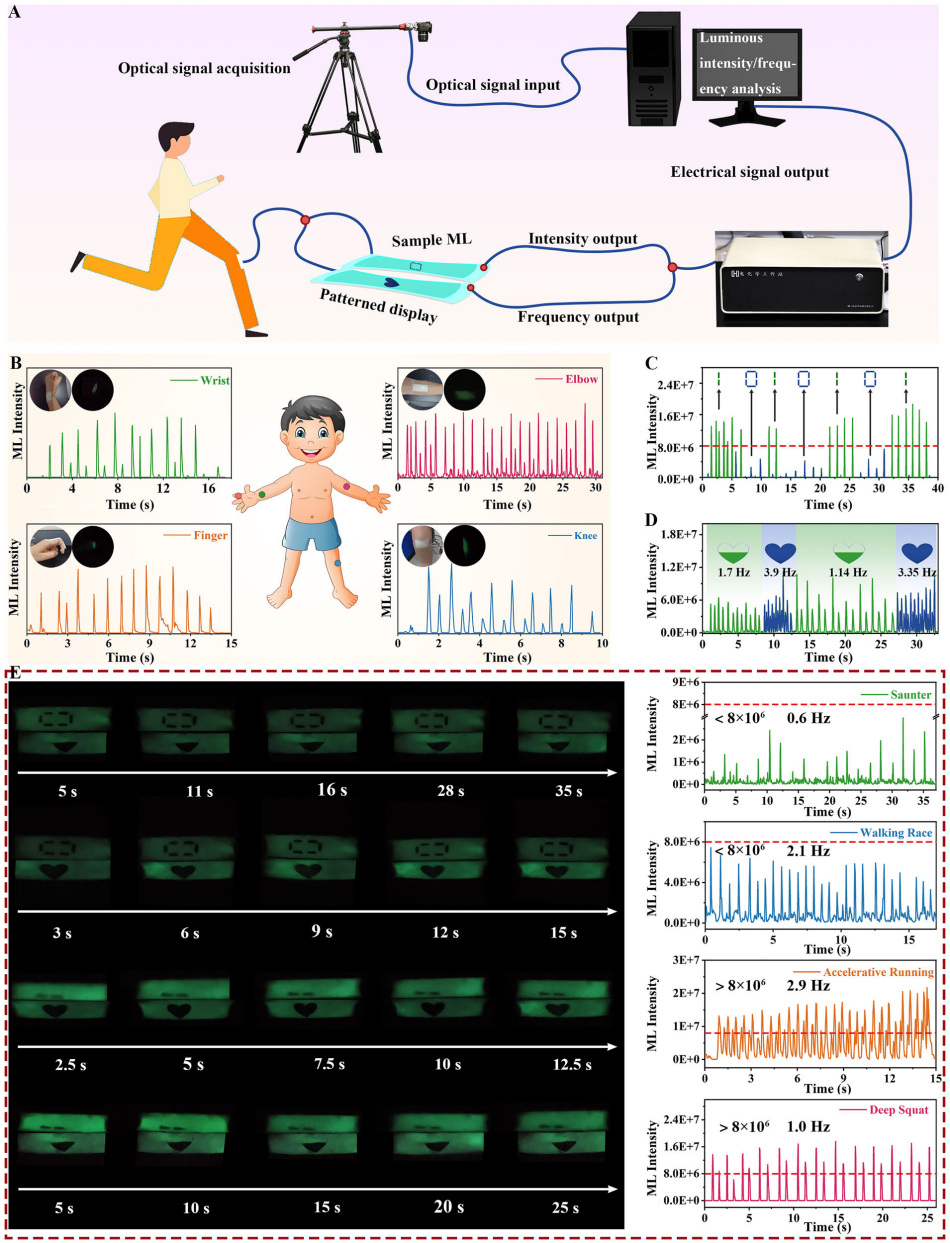
Multifunctional display applications for FMECDs. (A) Schematic diagram of FMECDs detection and display system. (B) ML strength of FMECDs when different body parts are bent (fingers, elbows, wrists and knees). (C) Data processing diagram of different stresses corresponding to the “0/1” signal. (D) Data processing diagram of “half 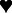
”/“ full 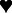
” signal corresponding to different drawing frequencies. (E) ML intensity and pattern displays of FMECDs in four different motion states (saunter, walking race, accelerative running, deep squat).

Different human motion states generate varying bending intensities and frequencies at joints. By monitoring these bending intensities and frequencies, it would be possible to effectively capture key data on human movement, facilitating the detection of motion across various body parts. Characteristic stress‐strain deformations result in the contact and separation between ZnS: Cu and PDMS, which in turn generate corresponding ML signals [27]. By analyzing the ML intensity and frequency, signals are translated into distinct electrical outputs by a computer. The ML signals of the FMECDs are captured using a custom measurement setup, comprising a camera, a signal acquisition computer, and an electrochemical workstation (Figure 5A). The ML light signal collected by the camera is digitized by the computer (refer to Experimental Section/Methods, Supporting Information), and the relationship between ML intensity and time is analyzed. When the device is attached to the flexible and moving body parts, such as fingers, elbows, wrists, and knees, it can effectively differentiate and monitor the various movement states (Figure 5B). Figure S22 shows the output of the ML output as the device is attached to fingers, wrists, elbows, and knees. The output of ML intensity is highest when the device is attached to fingers. The joints of fingers can provide a greater range of motion than the other body parts, allowing a higher degree of deformation for fingers, leading to bending and flexing easily.

Furthermore, we illustrate the detection of different movement states using a patterned display approach, offering a straightforward and direct visualization (Figure 5C,D). The system employs a tensile stress‐ML data model composed of binary indicators (0 and 1) and a frequency‐ML data model featuring symbols “half 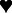
” and “full 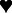
”. When the ML intensity surpasses a set threshold (>8 × 10^6^), the display shows a “1” signal; and below this threshold, it shows “0”. Similarly, when ML frequency exceeds 2 Hz, the display shows “full 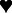
”; otherwise, it shows “half 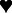
”. Here, the frequency of movement is defined by the number of repeated stretching changes made per unit time, in Hz. This pattern design relies on the direct correlation between ML intensity and applied strain (Figure S23, Supporting Information).The digital and pattern information of the electrically controlled FMECDs is shown in Figure S24 (Supporting Information). Notably, the wearable FMECDs demonstrate capability in monitoring body activities and displaying motion states, including saunter, walking race, accelerative running, deep squat (Figure 5E). According to pre‐determined correlations, FMECDs emit pattern signals corresponding to four motion states, represented by combinations of 0/1 and “half 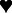
”/“full 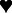
”. Taking the deep squat state as an example, the corresponding ML intensity is >8 × 10^6^, which is displayed by the digital signal “1”. The movement frequency is 1 Hz < 2 Hz, which is represented by the pattern signal “half 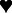
”. This proves that during human exercises involving greater tensile strength and slower movement speed, the individual is likely performing deep squats. This flexible, wearable electronic display offers potential applications in motion detection and pattern interaction interfaces, representing an advancement in multifunctional, portable, and scalable electronic devices for use in dark environments.

## 3 Conclusion

In summary, we demonstrate highly flexible, energy efficient FMECDs that are designed to work in dark environments and can be applied to patterned displays for human movement states. The FMECD integrates a stretchable ML‐PL substrate as the backlight source, enabling complementary EC and ML‐PL displays in dark environments. It shows a large optical modulation of 75.8% and stable mechanical and EC performance under 200 stretching‐recovery cycles (reflectance retention ≈ 86%) and 500 bending‐recovery cycles (reflectance retention ≈ 91.5%). Additionally, compared to commercial OLED displays, FMECDs achieve a significant reduction in energy consumption, down to 0.004%. This new device, which can be attached to flexible and movable parts of the human body, such as fingers, elbows, wrists, and knees, demonstrates the capability of distinguishing and monitoring the various body parts and motion states through patterned displays. The present study inspires the development of scalable flexible multi‐mode electrochromic displays and their applications in complex environments by integrating various optical materials with interactive components and computing terminals. In the future, FMECDs can be integrated into fiber‐based architectures compatible with textile fabrication, such as core–sheath electrochromic fibers or weavable luminescent yarns, for multifunctional wearable displays of highly deformable and washable.

## Conflicts of Interest

The authors declare no conflicts of interest.

## Supporting information




**Supporting File 1**: exp270155‐sup‐0001‐SuppMat.pdf.

**Supporting File 2**: exp270155‐sup‐0002‐MovieS1.mp4.

**Supporting File 3**: exp270155‐sup‐0003‐MovieS2.mp4.

## Data Availability

The data that support the findings of this study are available from the corresponding author upon reasonable request.
