# Supplementary material for: Flexible Multi‐Mode Electrochromic Displays for Human Motion Monitoring and Pattern Display in Dark Environments
Source: Exploration (Beijing). 2026 Mar 16;6(2):20240444. doi: 10.1002/EXP.20240444 (PMC13094525; doi:10.1002/EXP.20240444)
Supplement: Supplementary file 1 — Supporting File 1: exp270155‐sup‐0001‐SuppMat.pdf. [file EXP2-6-20240444-s003.pdf]

Supporting Information

Flexible Multi-mode Electrochromic Displays for Human Motion Monitoring and Pattern Display in Dark Environments

Xue Chen,[1] Qi Zhao[2], Li Wang,[1] Xin Yu,[1] Xu Liang,[1] Zhang Chen,[1*] John Wang,[2] Yanfeng Gao[1*]*

[1] School of Materials Science and Engineering, Shanghai University, Shanghai, China

[2] Department of Materials Science and Engineering, National University of Singapore, Singapore, Singapore

* Corresponding author. Email: yfgao@shu.edu.cn, zhaoqi95@nus.edu.sg, chenzhang@shu.edu.cn

1. Experimental Section/Methods

Preparation of surface pretreated PDMS flexible substrate: The flexible substrate was made of SYLGARD 184 SILICONE ELASTOMER, which included the basic component and curing agent (10:1 ratio). The mixed liquid was cured at 80°C for 25 minutes to obtain the elastic substrate PDMS. Then, tannic acid (TA) solution was used to pretreat PDMS.[1] First, the PDMS substrate was immersed in TA solution 5 (mg/mL) containing buffered brine (0.08 mol/L bicine, 0.5 mol/L NaCl) and stored for 24h under magnetic agitation. The modified PDMS was then removed from the solution, rinsed with rich deionized water, and dried in a nitrogen stream to obtain the modified PDMS (TA-PDMS) flexible substrate.

Solution process fabrication of silver nanowire (Ag NWs)@PEDOT:PSS-PDMS conducting electrodes: Silver nanowires (AgNWs) were prepared by an improved polyol method.[2] 12 mL of 0.35 mol $AgNO_3$/EG, 5 mL of 1.2 mmol $FeCl_3$/EG, and 88 mL of EG solution containing a mixture of 1.28 g PVP-1300000 was combined and heated in an oil bath at 140 °C while stirring at 160 rpm/min. The synthesis process continues until the color of the solution turned grey-silvery. The resulting Ag NWs were dispersed in anhydrous ethanol, achieving a concentration of 0.2 mg mL^{-1}. PEDOT: PSS solution was diluted to a 1:4 v/v ratio using deionized water. In a typical process, the PDMS was pre-stretched and fixed on the heating table. Then, the 2 mL AgNWs dispersion and 100 uL PEDOT: PSS dispersion were sprayed onto the PDMS with an air-brush, respectively. The distance between the nozzle and the PDMS was maintained at 15 cm. TA-PDMS stretches ahead to 50% of the shape variable.[3] The nozzle diameter was 0.5 mm, and the spraying pressure was 0.1 MPa. Finally, all samples were heated at 90 °C for 20 min.

Synthesis of WO_3 nanowires (WO_3 NWs): WO_3 NWs was synthesized based our previous work.[4] In a typical solvothermal process, 15 g $Na_2WO_4 \cdot 2H_2O$ was dissolved in 50 mL water with constant stirring. After complete dissolution, HCl was added to the solution until no more

precipitates were obtained. The precipitates were washed with water until pH=2 was reached. Then, precipitates were transferred to 80 mL EG with constant stirring at 80 °C until a transparent blue solution was obtained. The solution was transferred into a Teflon-lined autoclave with an internal volume of 20 mL for a solvothermal reaction run at 180 °C for 24 h. Finally, after washing with pure water, dark blue WO_3 NWs were obtained. 0.5 mL of AgNWs is sprayed onto the PDMS electrode and all samples were heated at 90°C for 20 minutes.

Preparation of the hydrogel electrolyte:[5] The liquid electrolyte was prepared by mixing 7.5M ZnCl in acrylamide (AM) aqueous solution (20% wt). The mother solution of the crosslinking agent was prepared by dissolving 0.06% wt N-N-monomethylene-bisacrylamide (MBAA) and 0.5% wt amine sulfite (APS) in 10 ml water. The photoinitiator mother liquor was prepared by dissolving 0.05% wt 2-ketoglutaric acid in 10 ml water. After that, 7.1ml liquid electrolyte, 560 ul crosslinker and 560 ul photoinitiator were uniformly mixed and poured into the mold, and cured in ultraviolet for 30 h to obtain Zn^{2+} hydrogel electrolyte.

Assembly of the FMECDs: The multifunctional flexible substrate with PL-ML effect was prepared by mixing and curing ZnS: Cu phosphor with liquid PDMS at the mass ratio of 1:2.5. FMECDs are assembled with the Ag NWs@PEDOT: PSS-WO_3 NWs film, the Zn electrode and Zn^{2+} hydrogel electrolyte, which were employed as the cathode, the anode and the hydrogel electrolyte. A wearable displays (5 cm × 10 cm, 2 cm × 6 cm) was prepared with special"SHU", numbers and "♥" patterns of chameleons.

Material characterization: The material structure and morphology were characterized by X-ray diffraction (XRD, Rigaku Ultima IV), X-ray photoelectron spectroscopy (XPS, Thermo Scientific K-Alpha), Fourier Transform Infrared Spectrometer (FTIR, Bruker Vertex 70) and field emission scanning electron microscopy (FESEM, Hitachi S4800).The optical transmittance of the films was characterized by a UV/Vis/NIR spectrophotometer (UH4150;

Hitachi, Japan). Electrochemical and electrochromic measurements were carried out in a quartz cell containing 0.2 M $Zn(ClO_4)_4$/PC on an electrochemical workstation (CHI760E, CHI Instruments, USA) using a three-electrode system. The hydrophilicity was tested by JCY contact Angle tester. Low temperature image was taken by FPR-A615 infrared imager. The PLE spectra were measured on the fluorescence spectrometer (Edinburgh Instruments, FLS 1000) with a Xe lamp. The ML spectra were collected using an ANDOR spectrometer (SR-500i-B1-R) equipped with an optical fiber and CCD camera (CCD-20259). The spectra of ML intensity change over time were processed by image J software.

2. Equation

Equation S1. The calculation of coloration efficiency (CE)

It can be calculated by the following equation:

$$CE=\triangle OD/\triangle Q=[\log(T_b/T_c)]/(Q/A) \quad (1)$$

where A is the surface area, and T_b and T_c refer to the transmittance of film in the bleached and colored states, respectively.

Equation S2. Areal capacitance

The areal capacitance is calculated according to the following equation:

$$C=I\Delta t/S\Delta V \quad (2)$$

where C (F cm^{-2}) is the areal capacitance, I (mA) represents the discharge current, and S (cm^2), ΔV (V) and Δt (s) designate the area of active materials, potential windows excluding the IR voltage drop and total discharge time, respectively.

Equation S3. The coloration energy

The energy consumption can be calculated according to the following equation:[6]

$$\mathbf{W/A = (I \int U(t)dt)/A} \quad (3)$$

where W is the energy consumption (mWh), A is the effective area (m^2), U is the voltage of the

EC device (V), I is the current generated in the EC device during coloring process (mA), t is the coloring time (s).

3. Supporting Figures

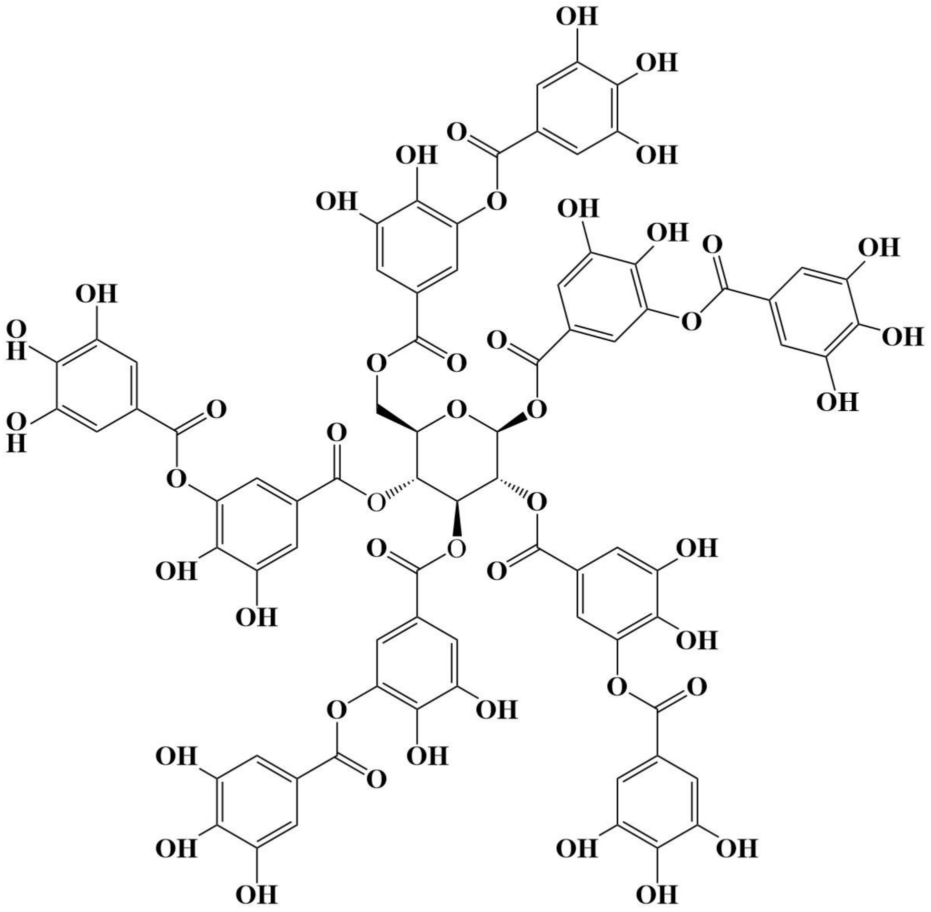

Figure S1. Molecular structure of tannic acid.

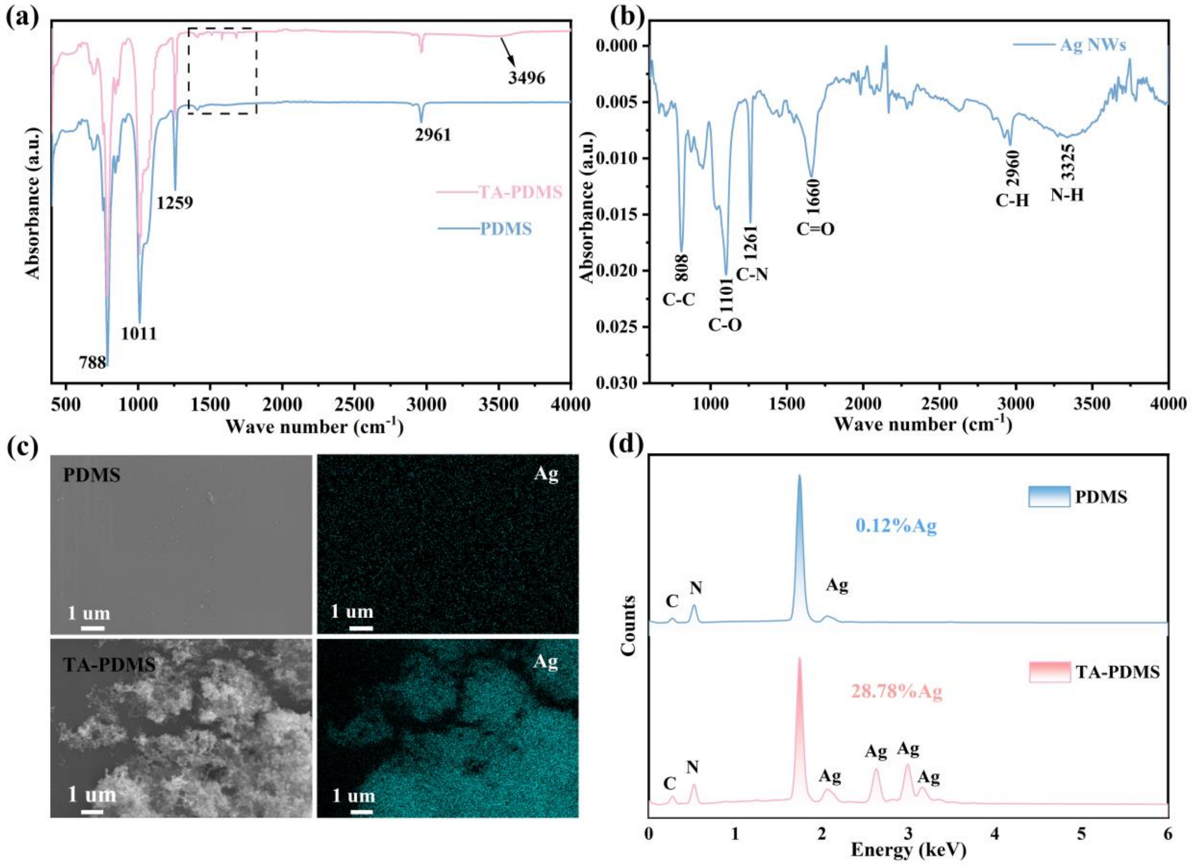

Figure S2. Surface characterization of TA modified PDMS flexible substrate. a) Fourier Transform Infrared Spectrometer (FTIR) characterization of untreated PDMS and TA-PDMS substrates. b) FTIR characterization of Ag NWs electrodes. c) SEM images and mapping of untreated PDMS and TA-PDMS substrate immersed in silver nitrate solution. d) Corresponding EDS element mapping results of the region.

Tannic acid (TA) has a large number of hydrophobic aromatic rings and hydrophilic phenolic hydroxyl groups (**Figure S1**), and it is easy to form hydrogen bonds with various molecules or groups, electrostatic, hydrophobic, π-π stacking and other interactions, which is very beneficial to the functionalization of the material surface interface.[7,8] As shown as in **Figure S2a**, Fourier Transform Infrared Spectrometer (FTIR) is used to characterize the untreated PDMS and TA modified PDMS substrates. In addition to PDMS intrinsic peaks at 789 cm^{-1}, 1011 cm^{-1}, 1259 cm^{-1} and 2961cm^{-1}, TA modified PDMS samples showed distinct bands at 1510 cm^{-1}, 1576 cm^{-1}, 1675 cm^{-1} and 3349 cm^{-1}.[9] The bands at 1510 cm^{-1} and 1576cm^{-1} are caused by the stretching

vibration of the benzene ring C=C in TA.[10] The substitution of hydrogen atoms in the benzene ring with electron-donating groups, such as hydroxyl groups,[10] the wide peak at 3496cm^{-1} is attributed to the stretching vibrations of the phenolic hydroxyl group. This analysis confirms that TA has been coated on the PDMS surface. In order to further clarify the reason why more Ag NWs can be adsorbed and deposited on TA modified PDMS substrate, we conducted FTIR tests on Ag NWs after drying, as shown in **Figure 2b**. Bands at 1261 cm^{-1}, 1660 cm^{-1}, 2960 cm^{-1} and 3325 cm^{-1} were generated by PVP during the synthesis of Ag NWs, and the characteristic peaks at 1660 cm^{-1} and 3325 cm^{-1} corresponded to the stretching vibration peaks of amide groups C=O and N=H in PVP. Hydrogen bond can be formed between the oxygen atom of amide group and the hydrogen atom of hydroxyl group on TA modified PDMS substrate, and the existence of hydrogen bond will greatly improve the adsorption and binding force of Ag NWs. To confirm the presence of TA in modified PDMS, we immersed both untreated and TA modified PDMS substrate in silver nanowire solution. As shown as **Figure S2c**, it is found that the untreated PDMS substrate presented a smooth surface with little Ag deposition. Conversely, a large amount of Ag is deposited on the surface of TA modified PDMS substrate after soaking in silver nanowire solution. The energy spectrum of the PDMS substrate before and after surface treatment is shown in **Figure S2d**. showing a significant increases in Ag content from 0.12% to 28.78%, which is mainly attributed to the chelation between Ag^+ and TA.[11,12]

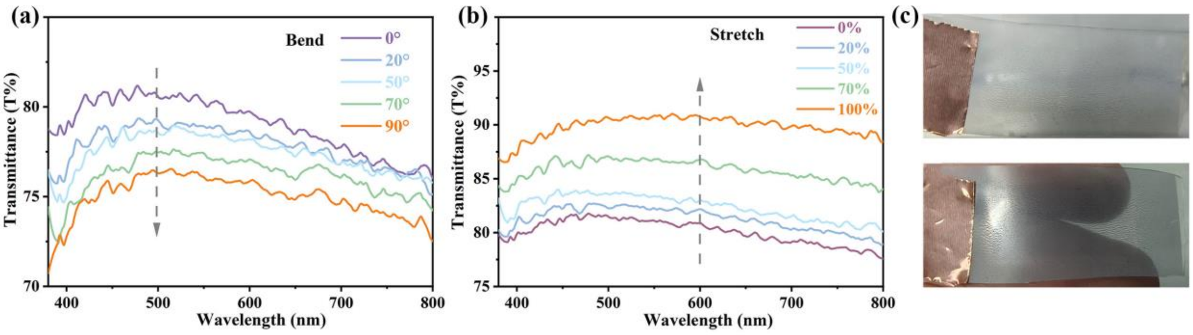

Figure S3. Transmittance of Ag NWs@PEDOT: PSS electrode under (a) bending and (b) stretching. (c) Photo of pleated electrode (top: Ag NWs; bottom: Ag NWs@PEDOT: PSS).

As shown in Figure S3, the test results show that as the stretching amount increases, the average transmittance of the electrode increases from 80% to 90% (380-800 nm). Conversely, as the bending angle increases, the average transmittance decreases to 75%.

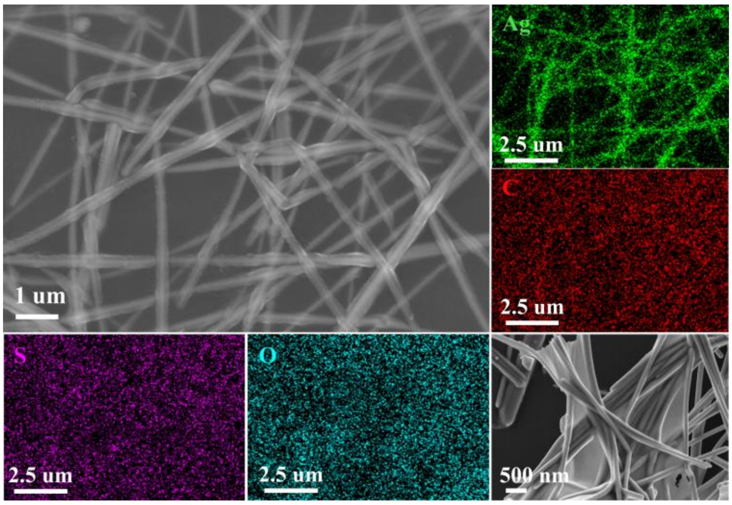

Figure S4. SEM images of Ag NWs@PEDOT:PSS.

Ag NWs@PEDOT: PSS conductive electrode is prepared on TA-PDMS by spraying method, as shown in **Figure S4**. In SEM image, it can be observed that AgNWs is wrapped in PEDOT: PSS, and and the C, O and S elements in the sprayed PEDOT are confirmed by mapping. The PSS layer is evenly distributed on the PDMS substrate and completely coated on the Ag NWs, which is conducive to the electrochemical protection of Ag NWs.

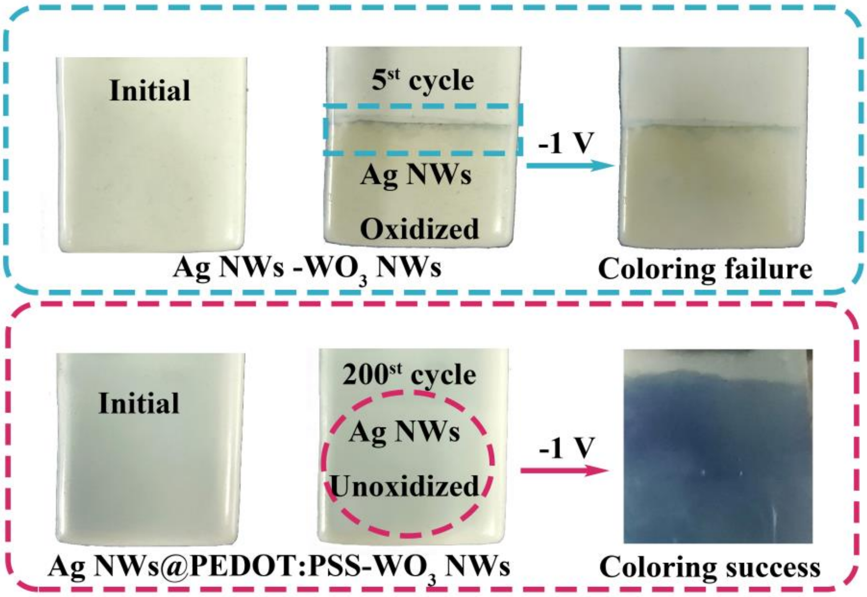

Figure S5. Photos without and containing PEDOT: PSS after a coloring/bleaching cycle test.

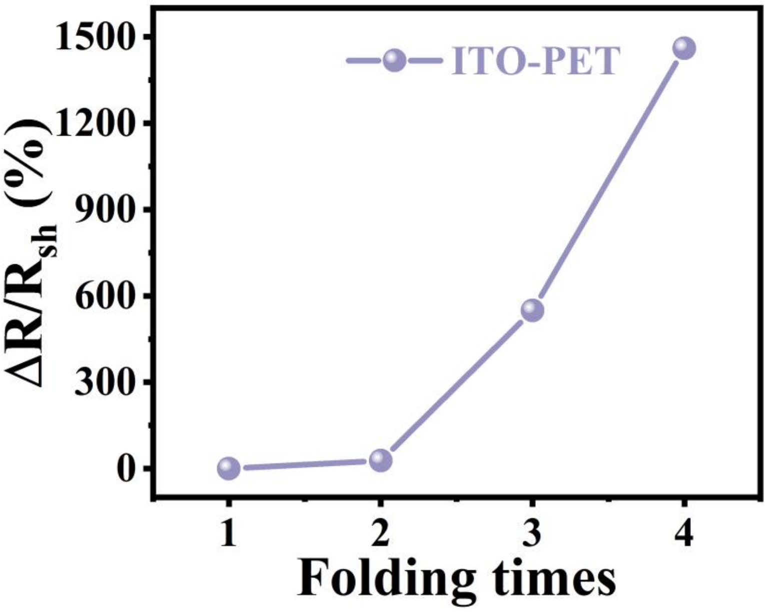

Figure S6. $\Delta R/R_{sh}$ diagram of ITO/PET electrode after bending.

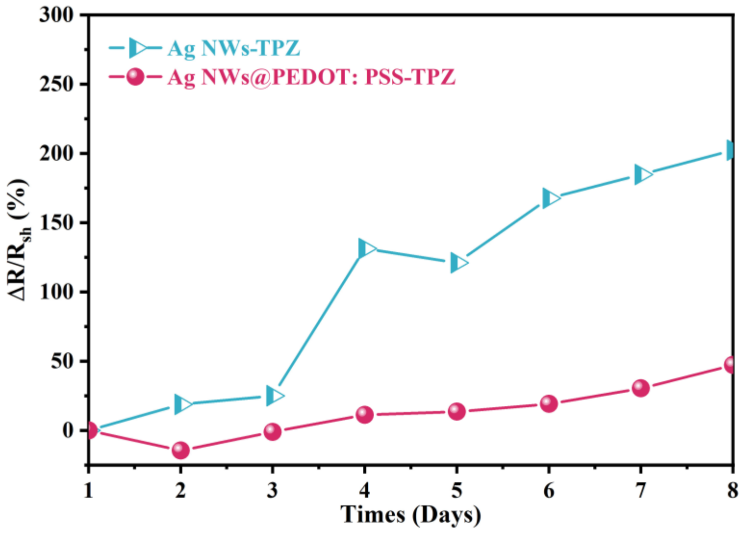

Figure S7. Long time stability of exposed Ag NWs-TPZ with Ag NWs@PEDOT: PSS-TPZ conductive substrate.

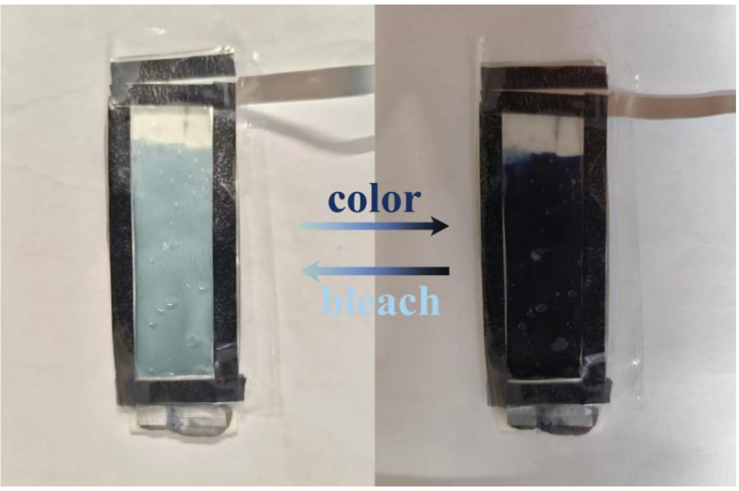

Figure S8. Physical diagram of FMECDs with Ag NWs@PEDOT: PSS-WO_3 electrode.

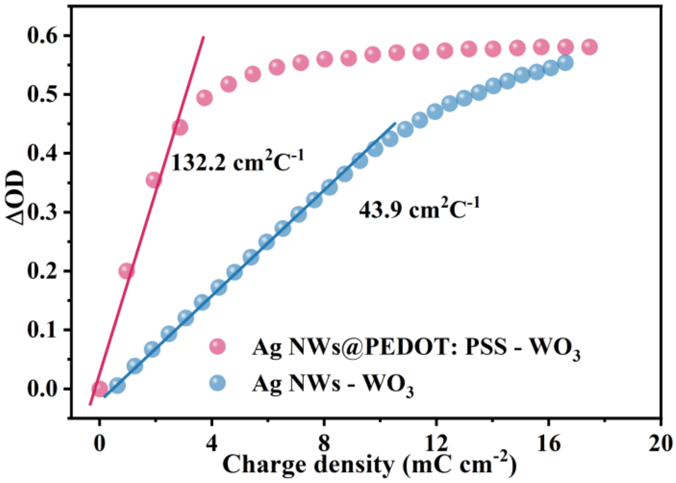

Figure S9. Coloration efficiency of FMECDs with Ag NWs-WO_3 and Ag NWs@PEDOT: PSS-WO_3 electrode.

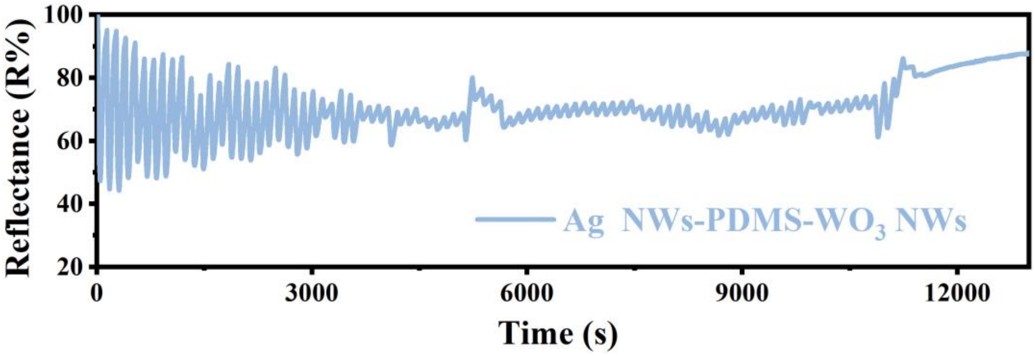

Figure S10. Cyclic stability of the Ag NWs-WO_3 device (200 cycles of coloring/bleaching).

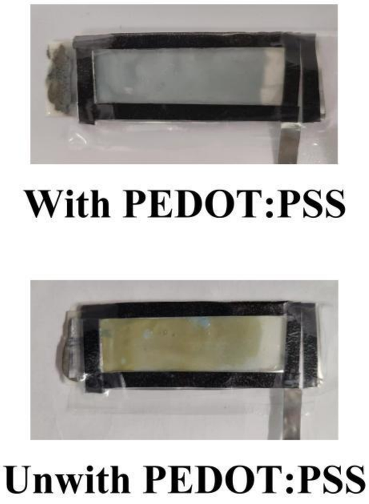

Figure S11. Physical drawings of FMECDs with (top) and without (bottom) PEDOT:PSS after 200

coloring/bleaching cycles.

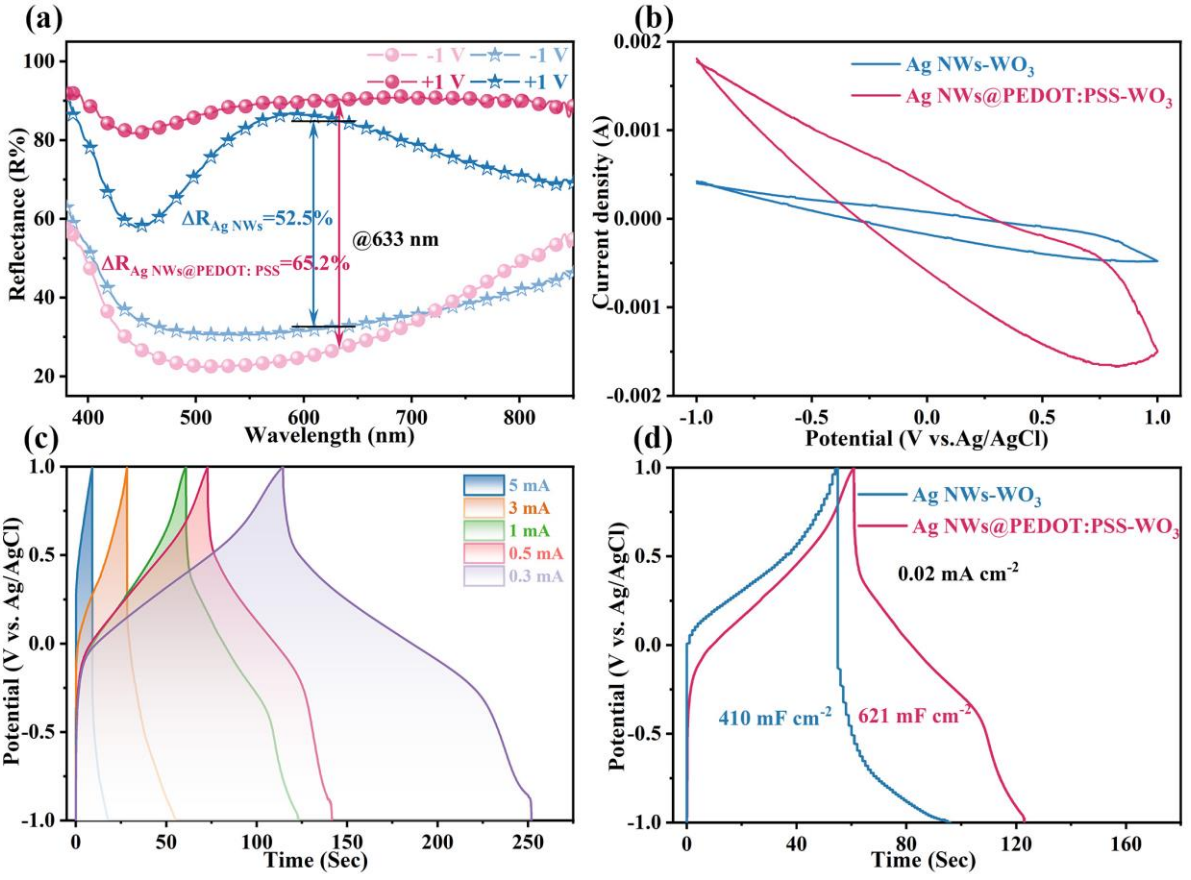

Figure S12. Ag NWs@PEDOT: PSS-WO_3 devices and Ag NWs-WO_3 devices a) Reflection spectra in colored/bleached conditions and b) CV curve at 0.1V s^{-1}. c) Galvanostatic charge/discharge (GCD) curve of Ag NWs@PEDOT: PSS-WO_3 electrode at various current densities. d) GCD curves of the Ag NWs@PEDOT: PSS-WO_3 electrode and the Ag NWS-WO_3 electrode at a current density of 0.02 mA cm^{-2} (the area capacitance of the electrode is 410 mF cm^{-2} and 621 mF cm^{-2}, respectively).

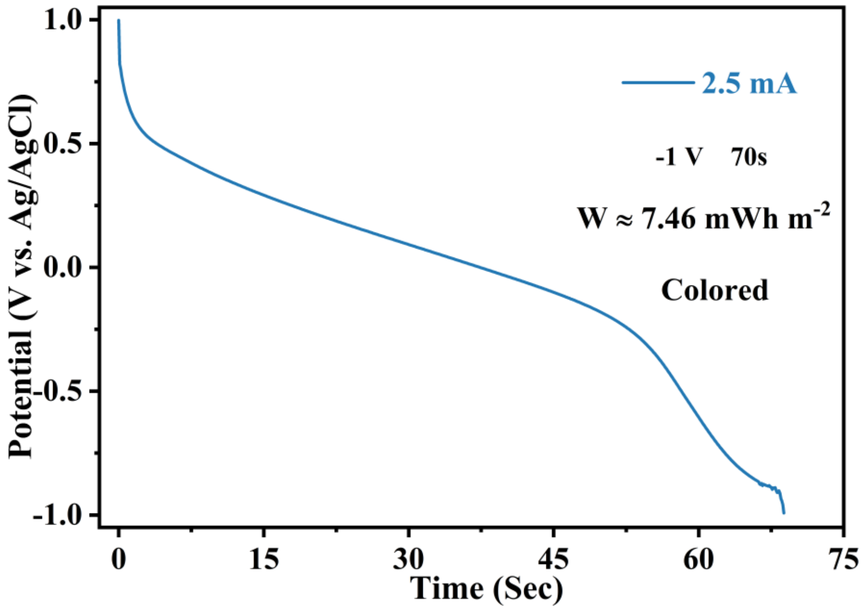

Figure S13. The Galvanostatic discharge curve at -1V, 70s has a current of 2.5mA, an effective size of 2.5 cm×3 cm, and a calculated energy consumption of 7.46 mWh m^{-2}.

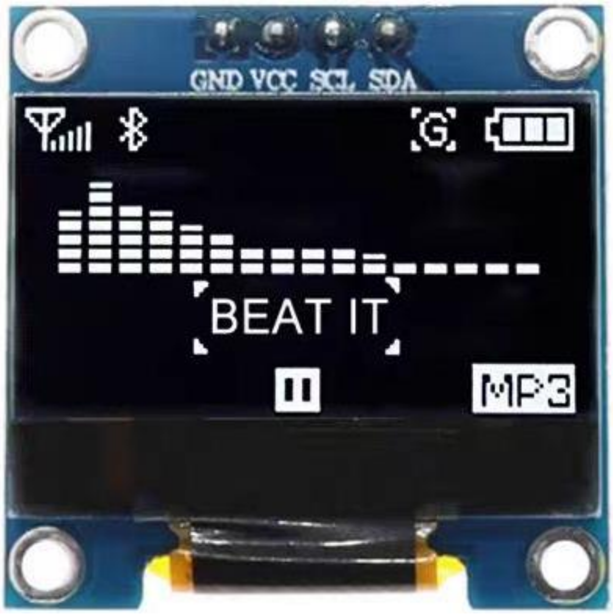

Figure S14. Schematic diagram of OLED display, where the power consumption is 21mA-28MAX and the operating voltage is 2.8/3.3V (The energy consumption for 1.5 hours is 172.19 Wh m^{-2}).

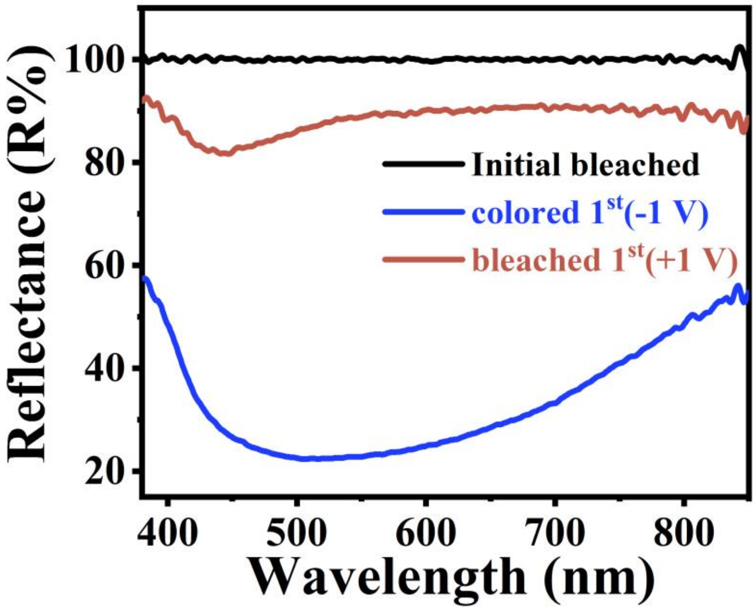

Figure S15. Normalized reflection spectrum of initial bleach state of FMECDs.

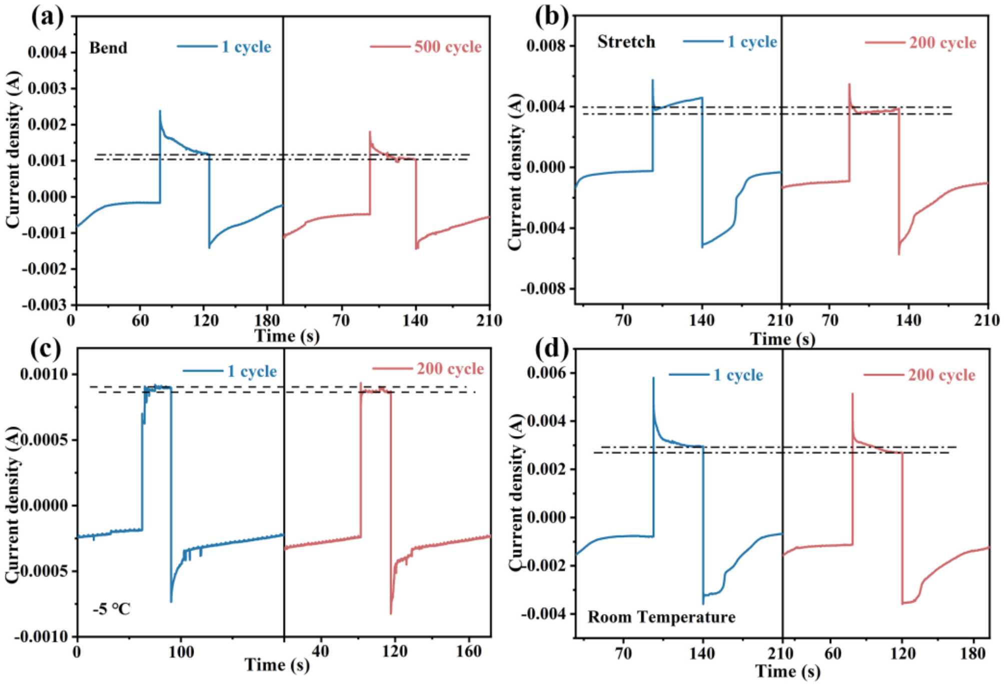

Figure S16. Characterization of the electrochemical stability of FMECDs at deformation, room temperature and low temperature.

Current-time curve of FMECDs was tested for 200 cycles of repeated stretching (50% strain)

and 500 cycles of repeated bending (180°) to verify the stability of their electrochromic (EC) performance (Figure S16a, b). Meanwhile, to further evaluate the anti-freezing properties of FMECDs in potential practical applications, the feasibility of FMECDs at -5℃ and room temperatures is verified by current-time curve and cycle stability (Figure S16c, d). The electrochemical stability of FMECD remains basically unchanged under long-term cycling conditions of mechanical deformation, low temperature and room temperature, ensuring the practicality of the device in complex environments.

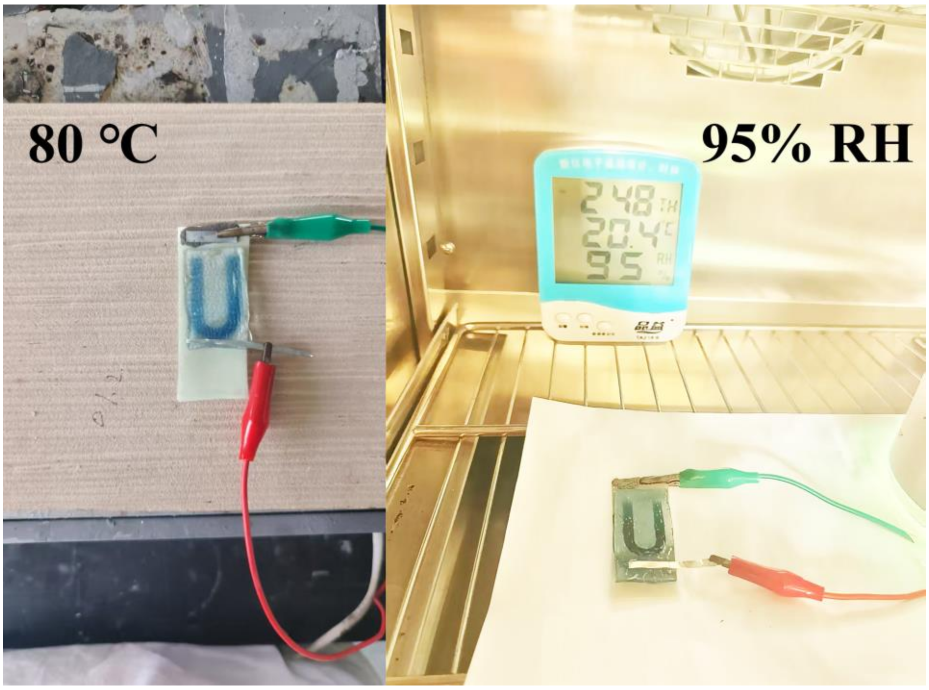

Figure S17. A physical picture of FMECD operating in a high-temperature/high-humidity environment.

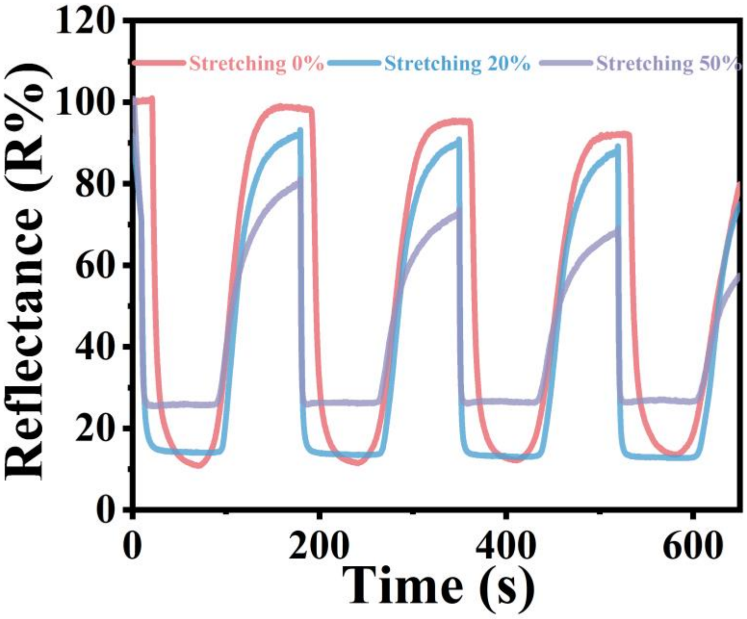

Figure S18. In situ reflectance spectra of Ag NWs-WO_3 devices under 20% and 50% tensile deformation.

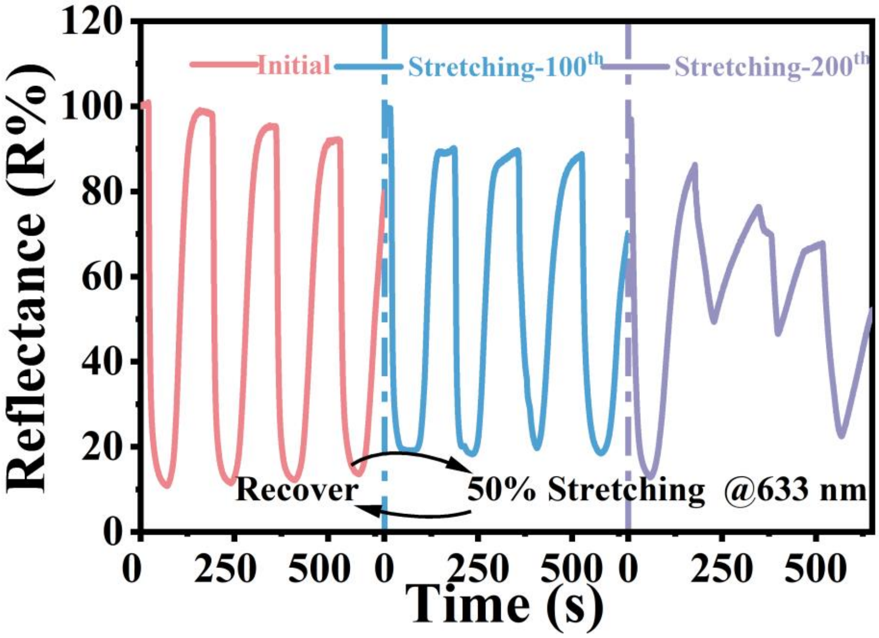

Figure S19. The Ag NWs-WO_3 device is repeatedly stretched (50% strain) for 200 cycles.

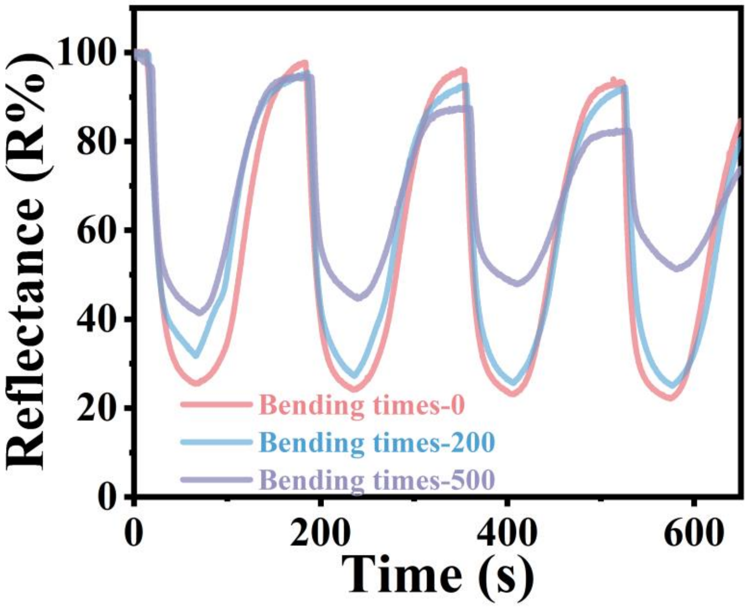

Figure S20. In situ reflectance spectrum of Ag NWs-WO_3 after 500 cycles of folding (180°).

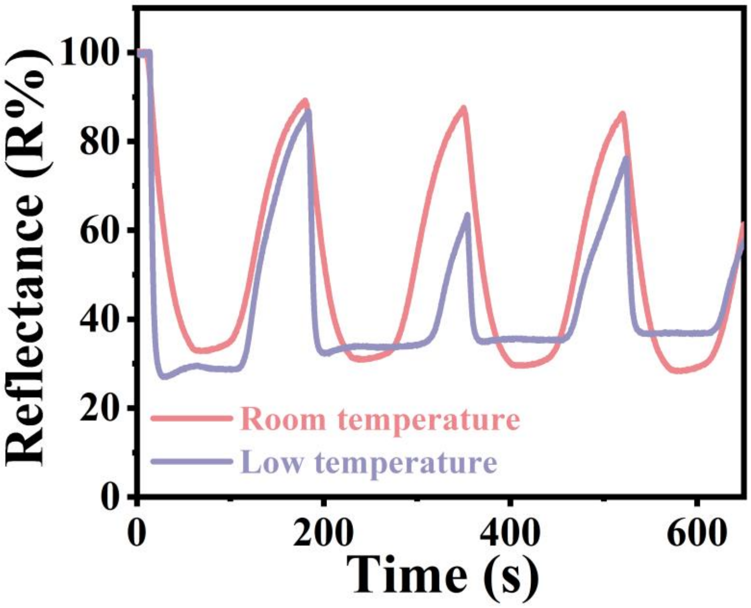

Figure S21. Low temperature stability of Ag NWs-WO_3.

As shown in **Figure S18-S21**, the electrochromic performance test of FMECDs prepared by Ag NWs-WO_3 electrode is presented. Compared with FMECDs with PEDOT:PSS protection,

devices without PEDOT:PSS protection have poor performance in repeated stretching, bending, low temperature, cycling and so on. **Figure S18** shows the image and reflectivity modulation amplitude test of the device under 20% and 50% tensile deformation. Compared with the device without tensile deformation, the modulation amplitude of 20% and 50% tensile deformation attenuates to the initial 63%. To verify the mechanical durability of the device, the device was repeatedly stretched (50% strain), folded (180°), and recovered for 200 and 500 cycles (**Figure S19** and **S20**). After repeated stretching 200 times, the contrast drops sharply and is unstable. The reflectance contrast test of the device after 180° folding shows that the reflectance modulation amplitude decreases to 53% after 200 and 500 bending times, and the large bending greatly affects the color display. **Figure S21** shows the low temperature stability, at low temperatures (-5°C), repeated coloring/bleaching occurs in an unstable state.

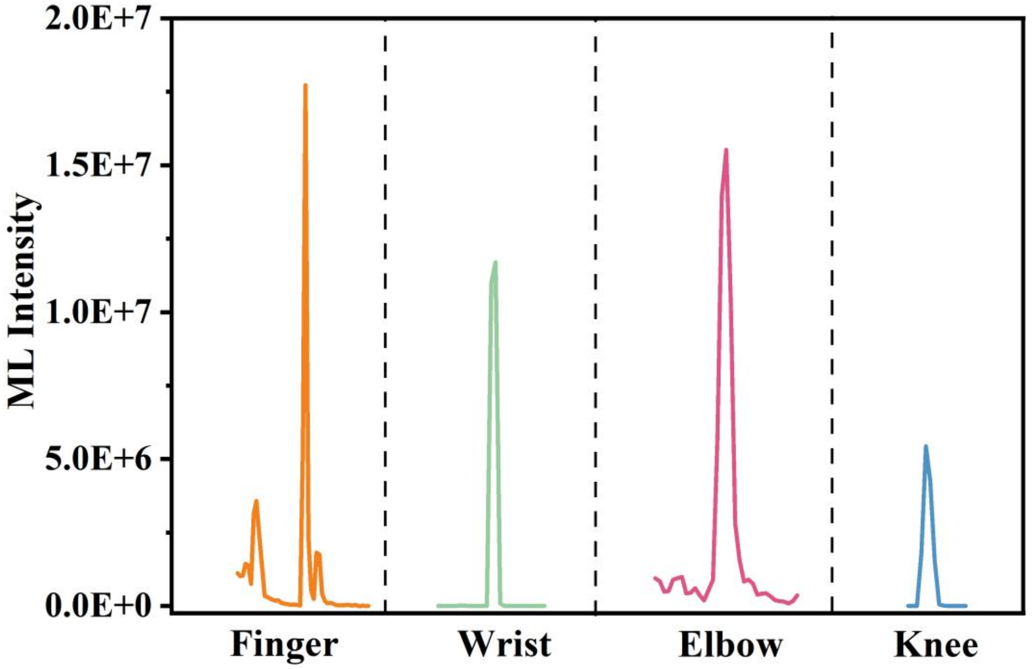

Figure S22. The ML intensity of the device when it is attached to different body parts (including fingers, wrist, elbow and knee).

Figure S22 shows the output of the ML output as the device is attached to fingers, wrists, elbows

and knees. The output of ML intensity is highest when the device is attached to fingers. The joints of fingers can provide a greater range of motion than the other body parts, allowing a higher degree of deformation for fingers leading to bending and flexing easily.

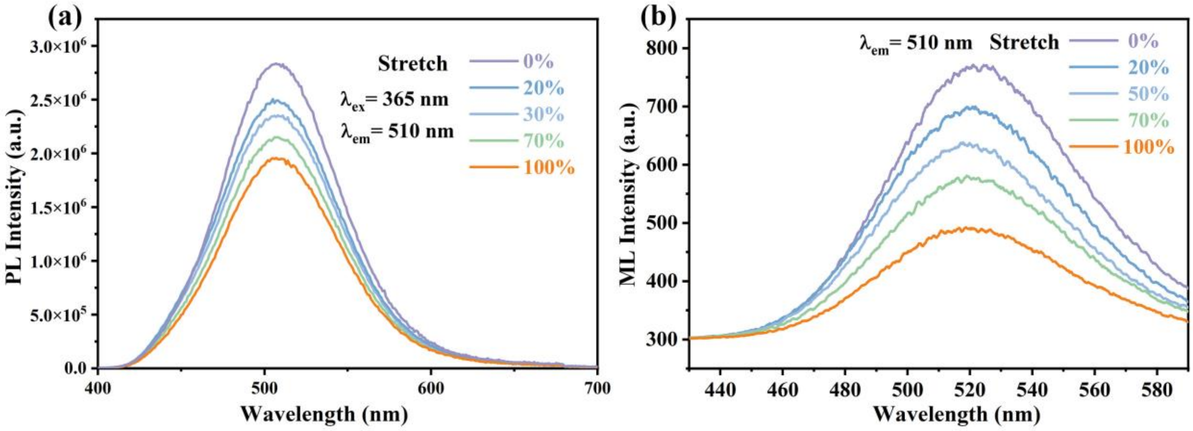

Figure S23. The luminescence intensities of (a) PL and (b) ML under different tensile states.

As shown in Figure S23, within the 0–100% stretching range, the PL intensity decreases with increasing strain, yet the luminescence remains above 70%, indicating that the composite structure has good deformation adaptability. The ML intensity, on the other hand, increases, as the elastic strain of the PDMS flexible substrate promotes localized stress accumulation within the ZnS lattice, which facilitates the conversion of energy into light emission. We have included this additional data in the supplementary materials for further analysis.

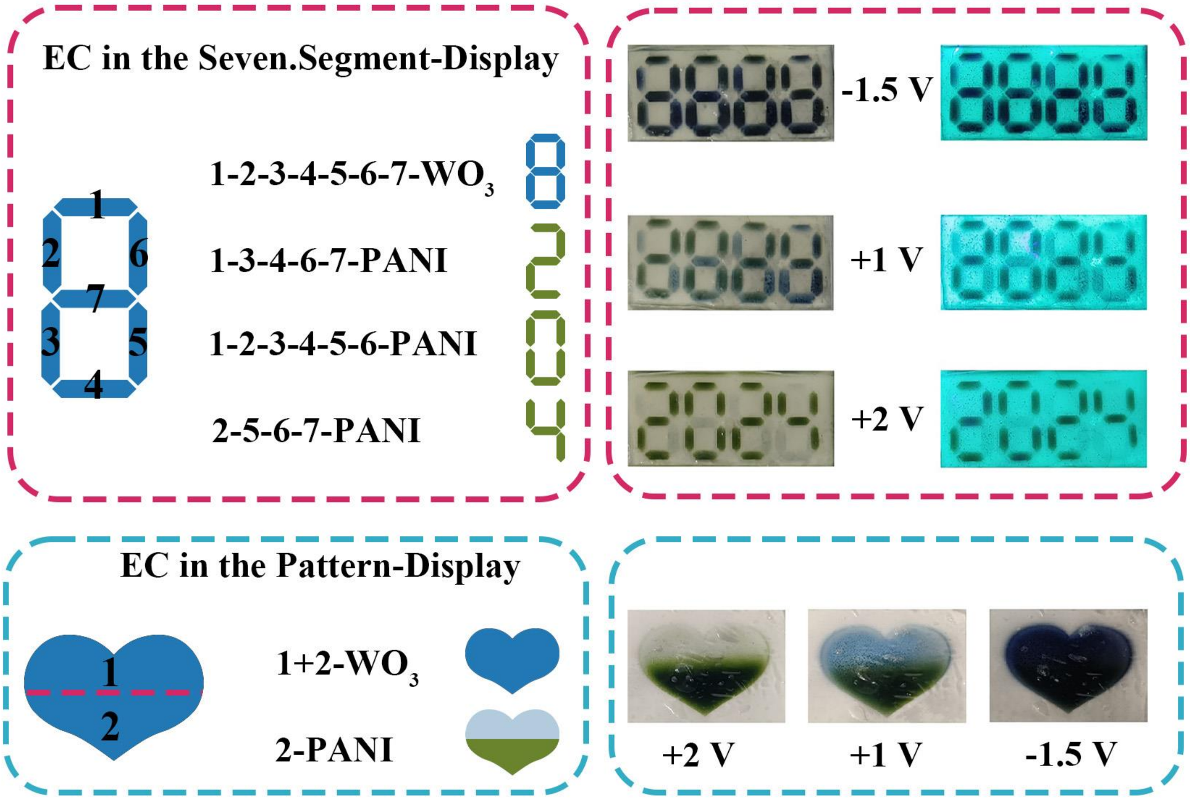

Figure S24. Digital and patterned information security and information storage for electronically controlled FMECDs (EC-PL mode). Digital design, using WO_3 spray "8888", in the polyaniline spray "2024". The conversion from 8888 to "2024" is achieved by applying a voltage of -1.5V to 2V. Pattern design, WO_3 is used to spray complete ♥, and the lower part of ♥ is sprayed with polyaniline. The conversion from "half ♥" to "full ♥" is achieved by applying a voltage of 2V to -1.5V.

4. Supporting Tables

Table S1. Summary of comparative data for previous articles and our work.

Display mechanism	Adaptation to lighting conditions	Mechanical testing	Energy consumption performance	Target application
Dual mode: EC+PL[13]	Rely on ambient light or external UV excitation	Stability under stretching	/	Flexible display device
Dual mode: EC+PL[14]	Rely on ambient light or external UV excitation	ITO-glass substrate, no deformation	/	Display in bright or dark conditions.
Dual mode: EC+EFC[15]	Rely on excitation	ITO-glass substrate, no deformation	/	Visualization of self-powered human movement under all-light conditions
Dual mode: EC+ECL[16]	Rely on Electricity excitation	ITO-glass substrate, no deformation	/	Display in bright or dark conditions.
Three modes: EC+PL+ML, introducing ML active backlighting for the first	Rely on active mechanical stress	Bending, stretching, low temperature and darkness	Extremely low energy consumption (0.0075 Wh m^{-2}), significantly superior to OLED	Wearable motion perception display, supporting dynamic state visualization under all-light conditions

time (this work)	(172.2 Wh m^{-2})

As summarized in Table S1, while prior studies have focused on electrochromic devices, our work introduces a multimodal display system integrating EC, PL, and ML, with an emphasis on evaluating stability under complex conditions such as darkness, mechanical deformation, low temperatures, and high humidity. Through a multimodal integration strategy, we demonstrate wearable motion sensing displays with dynamic state visualization, advancing the application potential of electrochromic technologies in next-generation flexible electronics.

Table S2. Summary of comparative data for Ag NWs@PEDOT: PSS electrode and other similar works.

Electrode Type	Resistance(Ω)	Transmissivity (%)	Stretching/Bending
Ag NWs [17]	440	92	10000 Bending
Ag NWs + Graphen [18]	212	84.5%	150% Stretching
Ag NWs + GO [19]	~10-20	/	/
Ag NWs + AZO [20]	~10-30	82.5	/
CNTs/ Graphene composites [21]	100	/	/
Ag NWs@PEDOT: PSS (This Work)	~10.8	92	150% Stretching

Compared to other commonly reported flexible transparent electrodes, the Ag NWs@PEDOT: PSS electrode developed in this work exhibits clear advantages. It achieves a low resistance (~10.8 Ω), which is superior to traditional Ag NWs networks and hybrid structures like Ag NWs/graphene composites. Moreover, it demonstrates outstanding stretchability up to 50%,

while maintaining high electrochemical stability under repeated bending and stretching (up to 500 cycles with minimal performance degradation). In contrast, other electrodes either suffer from higher resistance, limited stretchability, or poor long-term mechanical durability. Thus, the Ag NWs@PEDOT: PSS electrode provides a balanced combination of conductivity, flexibility, and mechanical robustness, making it highly suitable for use in flexible electrochromic devices.

Table S3 Summary of comparative data for previous articles and our work

Materials	ΔR/ΔT %	Coloring/bleach Time (s)	Cycling Life (Cycles)
WO_3 (This work)	75.8% (630 nm)	2.1/83.1	500 (bend)<br>200 (stretch)
$W_{18}O_{49}$/PET[22]	32% (630 nm)	4.1/8.5	500 (bend)
WO_3/PDMS[23]	40% (630 nm)	4.7s /4.5	200 (stretch)
Viologen/PET[24]	54.4% (360 nm)	15.3/10.4	5000 (bend)
PFPT/PET[25]	43% (850 nm)	0.9/3.1	5000 (bend)

As summarized in Table S3, the WO_3-based flexible electrochromic devices (FMECDs) developed in this work exhibit superior performance compared to previously reported systems. They achieve a high reflectivity modulation of 75.8% at 630 nm, fast coloration kinetics of 2.1 s, and robust mechanical durability over 500 bending cycles. This outstanding combination of optical tunability, rapid response, and flexibility highlights the significant advantages of our Ag NWs@PEDOT: PSS-based electrodes and their strong potential for practical applications in next-generation flexible displays and wearable electronics.

Table S4. The summary of electrochromic performance under bending conditions

Electrode	Bend (°)	ΔR/ΔT %	Response Time	Cycle Stability
FMECD (this work)	0	75.8	2.1s	500-91.5%
	180	74.2(200cycle)	4.6s(200cycle)	
		69.4(500cycle)	13s(500cycle)	
$W_{18}O_{49}$/PET	0	32	4.1s	500-75%
	180	24 (500cycle)	_	

Table S5. The summary of electrochromic performance under stretching conditions

Electrode	Stretch (%)	ΔR/ΔT %	Response Time	Cycle Stability
FMECD (this work)	0	75.8	2.1s	_
	20	73.3	3s	_
	50	71.7	3.7s	200-86%
WO_3/PDMS	0	40	2.5s	_
	20	30	4s	_
	50	27	4.7s	200-81%

Table S6. The summary of electrochromic performance under low-temperature conditions

Electrode	Temperature (℃)	ΔR/ΔT %	Response Time
FMECD	Room Temperature	68.1	2.1s
(this work)	-5	61.6	8s
WO_3/PET	-25	45	11.4s

Reference

[1] J. Zhao, *Chem. Res. Chin. Univ* **2019,** *35*, 5.

[2] Y. Ran, W. He, K. Wang, S. Ji, C. Ye, *Chem. Commun (Camb)* **2014**, *50*, 14877.

[3] B. Ji, M. Wang, C. Ge, Z. Xie, Z. Guo, W. Hong, X. Gu, L. Wang, Z. Yi, C. Jiang, B. Yang, X. Wang, X. Li, C. Li, J. Liu, *BIOSENS BIOELECTRON* **2019**, *135*, 181.

[4] Q. Zhao, J. Wang, X. Ai, Z. Pan, F. Xu, J. Wang, Y. Gao, *Nano Energy* **2021**, *89*, 106356.

[5] X. Ai, Q. Zhao, Y. Duan, Z. Chen, Z. Zhang, Y. Liu, Y. Gao, *Cell Rep. Phys. Sci.* **2022**, *3*, 101148.

[6] R. Fang, Z. Bai, X. Wu, Q. Fan, B. Bao, C. Hou, Q. Zhang, Y. Li, K. Li, H. Wang, *Adv. Opt. Mater* **2024**, *12*, 2400498.

[7] W. Yan, M. Shi, C. Dong, L. Liu, C. Gao, *Adv Colloid Interface Sci* **2020**, *284*, 102267.

[8] X. Fu, S. Yuan, F. Yang, H. Yu, Y. Xie, Y. Guo, W. Yao, *J Food Sci* **2023**, *88*, 1325.

[9] K. Chamerski, M. Lesniak, M. Sitarz, M. Stopa, J. Filipecki, *Spectrochim Acta A Mol Biomol Spectrosc* **2016**, *167*, 96.

[10] K. K. Pandey, *J. Appl. Polym. Sci* **1999**, *71*, 1969.

[11] B. Song, L. Yang, L. Han, L. Jia, *Materials (Basel)* **2019**, *12*, 1803.

[12] C. Yue, X. Qin, M. Hu, R. Zhang, B. Cheng, *REACT FUNCT POLYM* **2024**, *194*, 105798.

[13] W. Wu, W. C. Poh, J. Lv, S. Chen, D. Gao, F. Yu, H. Wang, H. Fang, H. Wang, P. S. Lee, *Adv. Energy Mater* **2023**, *13*, 2204103.

[14] W. Wu, H. Fang, L. Wu, H. Ma, H. Wang, *ACS Appl Mater Interfaces* **2023**, *15*, 4113.

[15] Y. Zhang, X. Jia, B. Sun, R. Huang, C. Wang, D. Chao, *Small* **2023**, *19*, e2301886.

[16] M. Pietsch, N. Casado, D. Mecerreyes, G. Hernandez-Sosa, *ACS Appl Mater Interfaces* **2022**, *14*, 43568.

[17] Y.-T. Kwon, J. W. Moon, Y.-M. Choi, S. Kim, S. H. Ryu, Y.-H. Choa, *J. Phys. Chem. C* **2017**, *121*, 5740.

[18] H. Bourahla, S. Fernández, Y. K. Ryu, A. Velasco, C. Malkia, A. Boscá, M. B. Gómez-Mancebo, F. Calle, J. Martinez, *Micromachines* **2025**, *16*, 204.

[19] S. Wang, Y. Feng, H. Zhang, Q. Peng, Y. Tian, *FLEX PRINT ELECTRON* **2021**, *6*, 024002.

[20] X. Yu, X. Yu, Y. Chen, T. Ji, S. Shen, T. Weng, M. Yan, L. Chen, Y. Zhou, J. Wei, *J. Alloys Compd* **2020**, *848*, 156569.

[21] H. Liu, D. Li, H. Liu, C. Wang, Y. Wang, Y. Chen, Y. Linghu, Z. Tian, H. Song, J. Zhou, L. Guo, *J. Colloid Interface Sci* **2022**, *620*, 168.

[22] Q. Zhao, J. Wang, J. Sun, C. Bao, X. Chen, J. Wang, Y. Liu, U. Bhat, C. H. Kirk, Y. Gao, *ENERGY ENVIRON MATER* **2024** *8*, e12790.

[23] S. Pereira, A. Gonçalves, N. Correia, J. Pinto, L. Pereira, R. Martins, E. Fortunato, *Sol. Energy Mater. Sol. Cells* **2014**, *120*, 109.

[24] X. Luo, R. Wan, Z. Zhang, M. Song, L. Yan, J. Xu, H. Yang, B. Lu, *Adv. Sci* **2024**, *11*, 2404679.

[25] Z. Wu, Q. Zhao, X. Luo, H. Ma, W. Zheng, J. Yu, Z. Zhang, K. Zhang, K. Qu, R. Yang, N. Jian, J. Hou, X. Liu, J. Xu, B. Lu, *Chem. Mater* **2022**, *34*, 9923.
